# The aged microenvironment impairs BCL6 and CD40L induction in CD4 ^+^ T follicular helper cell differentiation

**DOI:** 10.1111/acel.14140

**Published:** 2024-03-13

**Authors:** Jacob S. Fisher, Irene Adán‐Barrientos, Naveen R. Kumar, Jessica N. Lancaster

**Affiliations:** ^1^ Department of Immunology Mayo Clinic Scottsdale Arizona USA; ^2^ Immunobiology Laboratory Centro Nacional de Investigaciones Cardiovasculares (CNIC) Madrid Spain; ^3^ School of Life Sciences Arizona State University Tempe Arizona USA; ^4^ Robert and Arlene Kogod Center on Aging Mayo Clinic Rochester Minnesota USA; ^5^ Department of Cancer Biology Mayo Clinic Scottsdale Arizona USA

**Keywords:** aging, germinal center, T follicular helper cells

## Abstract

Weakened germinal center responses by the aged immune system result in diminished immunity against pathogens and reduced efficacy of vaccines. Prolonged contacts between activated B cells and CD4^+^ T cells are crucial to germinal center formation and T follicular helper cell (Tfh) differentiation, but it is unclear how aging impacts the quality of this interaction. Peptide immunization confirmed that aged mice have decreased expansion of antigen‐specific germinal center B cells and reduced antibody titers. Furthermore, aging was associated with accumulated Tfh cells, even in naïve mice. Despite increased numbers, aged Tfh had reduced expression of master transcription factor BCL6 and increased expression of the ectonucleotidase CD39. In vitro activation revealed that proliferative capacity was maintained in aged CD4^+^ T cells, but not the costimulatory molecule CD40L. When activated in vitro by aged antigen‐presenting cells, young CD4^+^ naïve T cells generated reduced numbers of activated cells with upregulated CD40L. To determine the contribution of cell‐extrinsic influences on antigen‐specific Tfh induction, young, antigen‐specific B and CD4^+^ T cells were adoptively transferred into aged hosts prior to peptide immunization. Transferred cells had reduced expansion and differentiation into germinal center B cell and Tfh and reduced antigen‐specific antibody titers when compared to young hosts. Young CD4^+^ T cells transferred aged hosts differentiated into Tfh cells with reduced PD‐1 and BCL6 expression, and increased CD39 expression, though they maintained their mitochondrial capacity. These results highlight the role of the lymphoid microenvironment in modulating CD4^+^ T cell differentiation, which contributes to impaired establishment and maintenance of germinal centers.

## Graphical abstract

Aging is associated with poor induction of germinal centers, organized lymphoid tissue structures that are important for infection and vaccination responses. CD4^+^ T cells provide help to B cells during germinal center induction, but the mechanisms by which they fail to support germinal centers are still unclear. Here we uncouple T cell‐intrinsic versus extrinsic changes with age that contribute to impaired CD4^+^ T‐cell activation and differentiation.

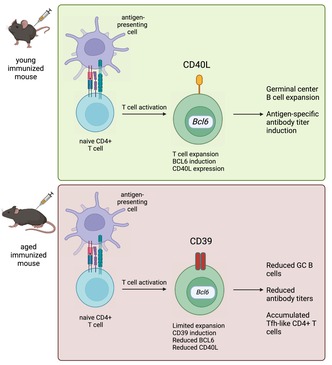

**Abbreviations**
APC: antigen presenting cell
cDC: conventional dendritic cell
GC: germinal center
MHCII: major histocompatibility complex II
NP: nitro‐phenyl hapten
OVA: ovalbumin
PBS: phosphate buffered saline
pDC: plasmacytoid dendritic cell
TCR: T cell receptor
Tfh: T follicular helper cell
Tfr: T follicular regulatory cell
Th1: type 1 helper T cell
Treg: regulatory T cell

## 1 INTRODUCTION

An effective immune response against infectious agents is characterized by the efficient induction of antigen‐specific lymphocyte responses, generation of high‐affinity antibodies, and subsequent establishment of immune memory. With aging, these key immune outputs are impaired, contributing to increased morbidity and mortality by acute viral and bacterial infections. Infectious disease is the fourth most common cause of death among older adults in developed nations, and 90% of excess deaths during regular flu seasons occur in people over 65 years old (Simon et al., 2015). Furthermore, dysfunctional immune responses with age directly result in subpar protection conferred by many vaccines (Ciabattini et al., 2018). This reduction in vaccine efficacy is especially significant for diseases with increased severity in older populations, such as in COVID‐19, in which humoral immunity waned significantly in older individuals by 3 months after vaccination (Doria‐Rose et al., 2021; Trigueros et al., 2022). While defects in immune outcomes with age have been characterized, the complex mechanisms driving immune dysfunction are still under investigation.

Long‐lived antibody‐mediated immunity is dependent on the formation of germinal centers (GCs) in response to immunization (Allen et al., 2007). GCs are distinct tissue structures that arise in the B cell follicles of the secondary lymphoid organs, the lymph nodes and white pulp of the spleen, and are regions of B cell proliferation and differentiation. The key immune outputs of GCs are plasma B cells, which generate class‐switched antibodies of high antigenic affinity, and memory B cells, which accelerate response rates to future re‐infection (Victora & Nussenzweig, 2022). GCs, which are well‐formed in infants and children, are often absent in histology samples from older adults (Luscieti et al., 1980). Immunization of aged mice similarly demonstrated poorly formed GCs with reduced antigen‐specific antibody titers in the serum (Richner et al., 2015). Thus, poor response to infectious disease in older individuals is likely linked to the failure to generate and maintain GCs.

CD4^+^ helper T cells play a prominent role in GC formation and function, and have also been implicated as being responsible for impaired GC responses with age (Eaton et al., 2004; Lefebvre et al., 2012). CD4^+^ T cells embark on a multi‐step journey during GC initiation, and it has been difficult to determine whether aging impairs T cells by inducing cell‐intrinsic changes, altering the roles of supportive cells or cytokines within the tissue microenvironment, or by some combination thereof. During infection, naïve CD4^+^ T cells are activated by cognate antigenic peptides presented by antigen‐presenting cells (APCs) within the T cell zone of the secondary lymphoid organs. A subset of activated CD4^+^ T cells upregulate the checkpoint molecule PD‐1, costimulator ICOS, and chemokine receptor CXCR5 as they migrate toward the B cell follicles, where they will interact with activated B cells via T cell receptor (TCR) engagements with major histocompatibility complex class II molecules presenting antigenic peptides (pMHCII) (Arnold et al., 2007; Liu et al., 2015; Shi et al., 2018). Prolonged interactions, stabilized by the binding of costimulatory CD40 ligand (CD40L;CD154) with CD40 on B cells, activate a differentiation program, guided by master transcription factor *Bcl6*, toward the T follicular helper cell (Tfh) fate (Liu et al., 2012). Tfh cells continue to migrate toward positions deeper within the follicle to the burgeoning GC, where they will serve their critical role in selecting GC B cells with high affinity for antigen. Diminished GC outcomes are difficult to reconcile with the apparent increase in Tfh cells observed in aging mice (Almanan et al., 2020; Sage et al., 2015). These studies, as well as analysis of circulating Tfh in human blood (Herati et al., 2021), have suggested that Tfhs have a different function within the aged GC context, though the mechanisms driving their expansion and their role in the aged humoral response are unclear.

In this study, we demonstrated that aging mice have accumulated cells with a Tfh‐like phenotype within the lymphoid tissues, which failed to upregulate BCL6 expression while exhibiting increased expression of ectonucleotidase CD39 with immunization. Aged Tfh cells also exhibited signs of mitochondrial impairment. In vitro, aged CD4^+^ T cells maintained proliferative capacity upon stimulation but had decreased upregulation of CD40L. Numbers of young CD4^+^ T cells upregulating CD40L were also reduced when activated by APCs derived from middle‐aged mice, indicating that T‐cell activity in aging can be altered by cell‐extrinsic means. Young, antigenspecific CD4^+^ T cells and B cells transferred into aged mice had reduced capacity for expansion and differentiation as compared to transfer into young hosts. The transferred CD4^+^ T cells differentiated into Tfh cells with reduced PD‐1 and BCL6 expression. However, cellular energetics appeared to be intrinsically wired in activated CD4^+^ T cells, as we did not observe signs of impaired mitochondrial function in young T cells exposed to the aged tissue microenvironment.

Thus, our results indicate that even short‐term exposure to the aged lymphoid microenvironment can alter some aspects of CD4^+^ T cell differentiation, supporting the role of cell‐extrinsic factors in limiting pathogen responses and vaccine efficacy in older organisms.

## 2 METHODS

### 2.1 Mice

C57BL/6J, B6.SJL‐Ptprca PepCb (CD45.1), B6.Cg‐Tg(TcraTcrb)425Cbn/J (OT‐II), and B^1‐8^; Jκ^−/−^ (A. Haberman, Yale University, New Haven, Connecticut, USA) were bred and maintained under specific pathogen‐free conditions in the Mayo Clinic animal facility. All strains were sourced from Jackson Laboratories, including aged C57BL/6J mice, unless specified. Within an immunization experiment, all mice were either uniformly male or female, with at least one experiment with each sex, which were combined for the results reported. Mouse maintenance and experimental procedures were carried out with approval from the Institutional Animal Care and Use Committee at the Mayo Clinic.

### 2.2 Immunizations and adoptive transfers

4‐Hydroxy‐3‐nitrophenylacetyl hapten conjugated to ovalbumin protein (NP‐OVA; Biosearch Technologies) was precipitated in aluminum potassium sulfate dodecahydrate (Acros Organics) for 3 h at 4°C at a concentration of 0.75 mg mL^−1^, and 100 μg per mouse injected intraperitoneally. For low dose injections, 77.4 μg per mouse was injected in the same volume of vehicle. For adoptive transfer experiments, naïve B cells were obtained from the spleens of B^1‐8^; Jκ^−/−^ mice by immunomagnetic purification with the EasySep Mouse B cell Isolation Kit (StemCell). Naïve CD4^+^ T cells were obtained from the spleens of OT‐II; CD45.1 mice by immunomagnetic purification with the EasySep Mouse Naïve CD4^+^ T cell Isolation Kit (StemCell). 3e6 B cells and 5e5 T cells per mouse were transferred intravenously by retro‐orbital or tail vein injection 24 h before immunization.

### 2.3 Flow cytometric analyses

Spleens were harvested at desired endpoints, mechanically disrupted, and filtered through 40‐μm nylon mesh. Red blood cells were lysed with ammonium‐chloride‐potassium (ACK) buffer before downstream analysis. Up to 10^7^ cells were incubated for 20 min on ice in 100 μL of phosphate‐buffered saline (PBS) + 2% fetal bovine serum (FBS) with fluorochrome‐conjugated antibodies directed against the mouse targets as shown in Table 1, diluted (1:200) from stock concentrations of 0.5 mg mL^−1^ unless otherwise noted. Antibody staining for CXCR5 was incubated for 1 h at 37°C. Intracellular staining with antibodies against BCL6 (1:100 dilution), FOXP3, and phosphorylated intracellular targets was achieved with FOXP3 staining kit (Invitrogen) per manufacturer's instructions. Phosphorylated targets were detected with Alexa Fluor 647 donkey anti‐rabbit IgG secondary antibody (Invitrogen A31573). Staining for hapten reactivity using NP‐PE (Biosearch Technologies) was incubated at a concentration of 5 μg mL^−1^ in PBS + 2% FBS for 20 min on ice. Staining for proliferation with 5 μM Cell Trace Violet (CTV; Invitrogen) in PBS was incubated for 20 min at 37°C in the dark. Mitochondrial activity was determined by staining live cells in diluted (1:40) MitoTracker Green (Cell Signaling Technology) and (1:100) Tetra‐methyl‐rhodamine ethyl ester (TMRE)‐TexasRed (BD Biosciences) from 1 mg mL^−1^ working solutions for 20 min at 37°C. Cells were co‐stained with Zombie Red (Biolegend) or Ghost Dye Red 780 (Tonbo Bioscience) at a concentration of 5 μg mL^−1^ with surface stain incubations or washed and resuspended in 1 μg mL^−1^ propidium iodide (PI) to determine viability. Cell numbers were quantified by automated counting (Corning CytoSmart device) or by the addition of 10‐μm polystyrene microspheres (Invitrogen). Samples were analyzed on an LSR Fortessa or Symphony flow cytometer (BD Biosciences) and data were analyzed using FlowJo (v.10, TreeStar).

**TABLE 1:** Anti‐mouse antibodies for flow cytometry.

Cell marker	Fluorophore	Clone	Vendor no.
CD3	BV421	17A2	Biolegend 100228
CD3	FITC	17A2	Biolegend 100204
CD3	APCCy7	17A2	Biolegend 100222
CD3	PECy7	17A2	Invitrogen 25‐0032‐82
CD4	AlexaFluor700	RM4‐5	BD Biosciences 557956
CD4	APC	GK15	Biolegend 100412
CD4	BV510	RM4‐5	Biolegend 100593
CD4	Unconjugated	GK1.5	BioXCell BE0003
CD8	BUV395	53–6.7	BD Horizon 563786
CD8	BV570	53–6.7	Biolegend 100739
CD8	Unconjugated	53.6.72	BioXCell BE0004
CD11b	AlexaFluor700	M1/70	Biolegend 101222
CD11b	APC	M1/70	Biolegend 101211
CD11c	BV711	N418	Biolegend 117349
CD11c	APC	N418	Biolegend 117310
CD19	APCCy7	6D5	Biolegend 115530
CD25	AlexaFluor700	PC61	Biolegend 102024
CD39	PEDazzle594	Duha59	Biolegend 143811
CD40	PE	3/23	BD Biosciences 553791
CD40L/CD154	APC	SA047C3	Biolegend 157009
CD40L/CD154	PE	MR1	Biolegend 106505
CD44	AlexaFluor700	IM7	Invitrogen 56‐0441‐82
CD45.1	PECy7	A20	Biolegend 110730
CD62L	BV605	MEL‐14	Biolegend 104437
CD69	APC	H1.2F3	BD Pharmingen 560689
CD69	PE	H1.2F3	BD Biosciences 553237
CD86	BV510	GL‐1	Biolegend 105039
B220/CD45R	eFluor450	RA3‐6B2	Thermo 48‐0452‐82
B220/CD45R	BV711	RA3‐6B2	Biolegend 103,255
B220/CD45R	Unconjugated	RA3.3A1/6.1	BioXCell BE006
BCL6	BV421	K112‐91	BD Horizon 563363
CXCR5	APC	L138D7	Biolegend 145506
CXCR5	BV650	LI38D7	Biolegend 145517
Fas	APC	SA367H8	Biolegend 152603
FOXP3	APC	FJK‐16S	eBioscience 17‐5773‐80
GL7	eFluor450	GL‐7	Invitrogen 48‐5902‐82
ICOS/CD278	PE	7E.17G9	eBioscience 12‐9942‐81
ICOS/CD278	PECy7	C398.4A	Biolegend 313519
Ly6C	BV570	HK1.4	Biolegend 128029
MHC‐II (I‐A/I‐E)	PECy7	M5/114.15.2	Biolegend 107629
NK1.1	BV421	PK136	eBioscience 48‐5941‐1630
NK1.1	Unconjugated	PK136	BioXCell BE0036
pAkt308 (Thr308)	Unconjugated	D25E6	Cell Signaling 13038T
pAkt473 (Ser473)	Unconjugated	Polyclonal	Cell Signaling 9271T
pAMPK (Ser79)	Unconjugated	Polyclonal	Cell Signaling 3661S
PDCA‐1/CD317	FITC	927	Biolegend 127007
pS6 (Ser 235/236)	Unconjugated	Polyclonal	Cell Signaling 2211S
PD‐1/CD279	eFluor450	RMPI‐30	eBioscience 48‐9981‐82
PD‐1/CD279	FITC	29F.1A12	Biolegend 135213
PSGL‐1/CD162	BV605	2PH1	BD Biosciences 740384
T‐BET	PECy7	4B10	Biolegend 644823
TCR Vα2	APCCy7	B20.1	Biolegend 127818
TCR Vβ5	FITC	MR9‐4	Biolegend 139514
XCR1	APCCy7	ZET	Biolegend 148223

### 2.4 Serum antibody titers

96‐well flat‐bottom plates were incubated overnight at 4°C with (1:250) 4 μg mL^−1^ of NPbovine serum albumin (BSA) (Biosearch Technologies). After wash with PBS with 0.05% Tween20 (washing buffer), wells were blocked with PBS with 10% FBS at 23°C for 1 h. Sample were incubated at 23°C for 2 h before probing with biotinylated rat anti‐mouse IgG1 (A85‐1; BD Biosciences 553,441) diluted (1:1000) from 1 μL mL^−1^ stock concentration with streptavidin horseradish peroxidase (BD Biosciences; 51‐75477E) diluted (1:250) from 4 μL mL^−1^ stock concentration at 23°C for 1 h. Peroxidase activity was developed with TMB substrate solution (eBioscience; 00–4201‐56) at 23°C for 0.5 h and reaction stopped with addition of 4 N sulfuric acid. Absorbance at 450 nm was measured with a SynergyHTX plate reader (BioTek). End‐point titers were determined using Prism software (GraphPad Software) from a one‐phase exponential decay curve defined as the dilution that generates an OD_450_ absorbance value of the background plus three standard deviations (Liu et al., 2012).

### 2.5 T‐cell activation assays

96‐well flat bottom plates were incubated overnight at 4°C with 2 ng mL^−1^ of purified antibody against mouse CD3 (145‐2C11; BD Biosciences 553,058) and 0.8 μg mL^−1^ of purified antibody against mouse CD28 (37.51; BD Biosciences 553,295) in PBS. For CD28 mobilization assay, flat bottom plates were prepared in the same manner, omitting CD28. 4e5 CTV‐labeled naïve CD4^+^ T cells isolated by immunomagnetic purification were cultured in each well for 24–72 h in complete RPMI 1640 medium [RPMI 1640 (Corning) and 10% fetal bovine serum (Gibco), supplemented with 100 U mL^−1^ penicillin–streptomycin (Thermo Fisher), non‐essential amino acid solution (Sigma Aldrich), 1 mM sodium pyruvate, GlutaMAX (Thermo Fisher), and β‐mercaptoethanol (Sigma Aldrich)] at 37°C/5% CO_2_. Cells were collected from wells by repeated pooled washings of PBS + 2% FBS before flow analyses.

For antigen‐presentation assays, bulk splenocytes (APCs) after ACK lysis were antigenpulsed by incubation in complete RPMI medium with varied concentrations of OVAp_323‐339_ (Genscript) for 1 h at 37°C. In other experiments, T, B, and NK cells were depleted from bulk splenocytes that were concentrated to 2e8 cells mL^−1^ in of PBS + 2% FBS and incubated in 48 μg mL^−1^ of antibodies against CD3, CD4, CD8, B220 and 5 μg mL^−1^ of antibody against NK1.1 (Table 1) for 30 min at 4°C, followed by immunomagnetic depletion using sheep anti‐rat IgG magnetic DynaBeads (Life Technologies) at a 4:1 cell: bead ratio. Magnetic depletion was repeated with half the number of beads to improve enrichments (yield ≥90% CD4^−^CD8^−^B220^−^NK1.1^−^) before pulsing with OVAp_323‐339_. Depletion of CD4^+^ and CD8^+^ T cells, NK1.1^+^ NK cells, and B220^+^ B cells was assessed by flow cytometry, with the presence of antibody‐bound yet undepleted cells detected with goat α‐rat IgG‐AlexaFluor488 (Poly4054; Biolegend 405418). 2.5e6 bulk APCs (5:1 ratio) or 1e6 T/B/NK‐depleted APCs (2:1 ratio) were co‐cultured with 5e5 naïve CD4^+^ T cells purified from OT‐II; CD45.1 mice in 96‐well round‐bottom plates in complete RPMI medium for 48 h. Cells were collected from wells by repeated pooled washings of PBS + 2% FBS before flow analyses.

### 2.6 Quantitative PCR

RNA from up to 1e7 cells was purified using the RNeasy Mini Kit (Qiagen, cat. no. 74104) according to manufacturer's instructions, and RNA concentration was then measured by NanoDrop One (Thermo Scientific). cDNA was synthesized using SuperScript® IV First‐Strand Synthesis System (Invitrogen, cat. no. 18091050) per the manufacturer's instructions, on a C1000 Touch thermocycler (BioRad). Gene expression levels of *Il6*, *Il21*, *Tgfb1*, and *Baff* were measured relative to *Actb* using PowerTrack™ SYBR™ Green Master Mix (Applied Biosystems, cat. no. A46109) on a Quantstudio™ 7 Pro Real‐Time PCR System (ThermoFisher Scientific, cat. no. A43183) following the manufacturer's instructions, using the following primers: *Actb*: forward‐CATTGCTGACAGGATGCAGAAGG, reverse‐TGCTGGAAGGTGGACAGTGAGG; *Il6*: forward‐ACGGCCTTCCCTACTTCACA, reverse‐CATTTCCACGATTTCCCAGA; *Il21*: forward‐CGCCTCCTGATTAGACTTCG, reverse‐TGGGTGTCCTTTTCTCATACG (Simard et al., 2011); *Tgfb1*: forward‐TGATACGCCTGAGTGGCTGTCT, reverse‐CACAAGAGCAGTGAGCGCTGAA; *Baff*: forward‐ AGGCTGGAAGAAGGAGATGAG, reverse‐CAGAGAAGACGAGGGAAGGG (Giordano et al., 2020).

### 2.7 Statistics

All statistical analyses were performed using Prism (v. 10, GraphPad) with the corresponding test listed in the figure legends.

## 3 RESULTS

### 3.1 Defective GC B cell expansion in aged mice is associated with abundant CD4 ^+^ T follicular helper cells with reduced BCL6 expression

**FIGURE 1:**
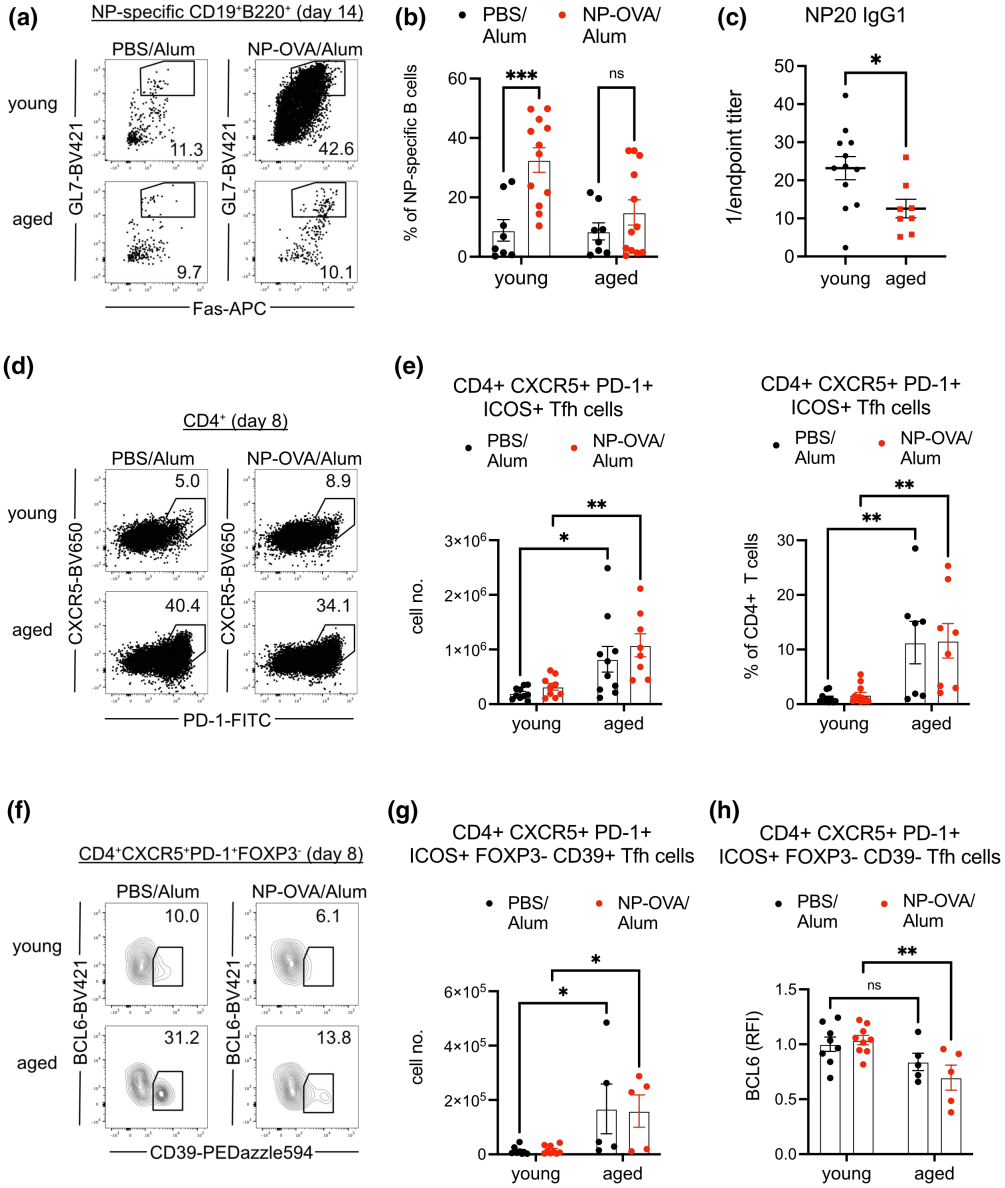
Defective GC B cell expansion in aged mice is associated with abundant CD4^+^ T follicular helper cells with reduced BCL6 expression. (a) Young (2‐ to 3‐month‐old) and aged (1822‐month‐old) mice were immunized with NP‐OVA/Alum or PBS/Alum, and the differentiation of NP‐specific GL7^+^Fas^+^ GC B cells was analyzed 14 days post‐immunization (dpi). (b) Frequencies of GL7^+^Fas^+^ GC B cells within NP‐specific B cell population in young and aged mice 14 dpi. (c) Endpoint titers of NP20‐specific IgG1 antibodies from sera of immunized young and aged mice 14 dpi. (d) CD4^+^ T cells in young and aged mice immunized with NP‐OVA/Alum or PBS/Alum were analyzed at 8 dpi. (e) Total CD4^+^CXCR5^+^PD‐1^+^ICOS^+^ Tfh cell numbers and their frequencies within CD4^+^ T cells of young and aged mice 8 dpi. (f) BCL6 versus CD39 expression on CD4^+^CXCR5^+^PD‐1^+^FOXP3^−^ Tfh cells of young and aged mice 8 dpi. (g) Numbers of CD4^+^CXCR5^+^PD‐1^+^ICOS^+^FOXP3^−^ Tfh cells that express CD39. (h) Relative fluorescence intensities (RFI) of BCL6 on CD39^−^ Tfh cells of young and aged mice 8 dpi, normalized to young mice treated with PBS/Alum. Data compiled from three experiments with each data point representing a mouse, bars represent mean ± SEM. Tested by two‐way ANOVA with Sidak correction for multiple comparisons, *p*‐values: *** < 0.001, ** < 0.01, * < 0.05, ns: not significant.

Young (2‐3‐month‐old) and aged (18‐22‐month‐old) mice were immunized via intraperitoneal injection with nitro‐phenyl hapten conjugated to ovalbumin (NP‐OVA) emulsified in alum adjuvant. Analysis of GC B cells, gated within B220^+^CD19^+^ lymphocytes (Rodig et al., 2005; Wang & Carter, 2005; Yazicioglu et al., 2023), revealed diminished expansion of NP‐specific GL7^+^Fas^+^ GC B cells in aged mice at 14 days postimmunization (dpi) as compared to young mice (Figure S1A, Figure 1a,b), which corresponded to reduced recovery of NP‐specific IgG1 from aged mouse serum (Figure 1c). We administered a standard antigen dose to all mice, as others have done when administering NP‐OVA to either aged (Almanan et al., 2020; Lefebvre et al., 2012; Silva‐Cayetano et al., 2023) or obese mice (Deng et al., 2021). Noting that aged mice in our colony were greater in mass than young mice (27.4 ± 2.7 g vs. 21.2 ± 0.7 g), we conducted a trial in which a set of young mice received a 23% lower dose of NP‐OVA/Alum. Ultimately, we found that young mice receiving reduced NP‐OVA/Alum still generated comparable frequencies of NP‐specific GC B cells as those with the standard dose (Figure S1B). This suggested that changes to antigen‐specific GC B cell induction were inherent to the aged mouse, rather than reduced antigen exposure because of increased body mass.

Polyclonal CD4^+^ T cells, analyzed 8 dpi, responded to peptide immunization by contracting their numbers and frequency within the spleens of aged mice (Figure S2A). Within the CD4^+^ T cell population, PD‐1^−^CXCR5^−^FOXP3^−^ conventional T cells (Tconvs) were decreased in numbers and abundance in both control and immunized aged mice as compared to young mice (Figure S2B,C). On the other hand, cell numbers of FOXP3^+^CD4^+^ regulatory T cells (Tregs) were maintained with age, driving increased frequency of Tregs in immunized aged mice as the Tconv compartment had decreased (Figure S2D,E). While increased Treg frequency in aged murine secondary lymphoid organs is in agreement with previous literature (Srinivasan et al., 2021), we found that the aged Tregs had decreased FOXP3 expression (Figure S2F), unlike what has been reported by others (Lages et al., 2008). We did find the aged Tregs had increased PD‐1 expression (Figure S2G), in agreement with a recent study (Sage et al., 2015). Quantification of CXCR5^+^PD‐1^+^ICOS^+^CD4^+^ Tfh revealed greatly increased numbers and frequencies in aged mice, even in unimmunized controls (Figure 1d,e), similar to what others have reported (Almanan et al., 2020; Sage et al., 2015). To confirm Tfh identity, we stained for intracellular BCL6 (Figure 1f), the master Tfh transcription factor (Liu et al., 2012). We also analyzed surface expression of CD39 (Figure 1f), a purinergic signaling modifier that negatively regulates *Bcl6* and is known to be increased in older individuals (Cao et al., 2020). Both naïve and immunized aged mice had greatly increased numbers of CD39^+^ Tfh cells (Figure 1g). CD39^+^ Tfh were clearly BCL6^lo^ (Figure 1f), and even after gating out CD39^+^ cells, we found that BCL6 expression was reduced in immunized aged mice when compared to young mice (Figure 1h). Finally, we quantified T follicular regulatory (Tfr) cells by gating for CXCR5^+^PD‐1^+^ICOS^+^FOXP3^+^CD4^+^ cells. Like Tregs and Tfhs, Tfrs were also greatly increased in numbers and frequencies in both control and immunized aged mice (Figure S2H,I). Our analysis demonstrates that aging is associated with increased Tfh‐like cells with decreased levels of BCL6 induction.

### 3.2 Intracellular signaling downstream of TCR is impaired in aged Tfh cells

**FIGURE 2:**
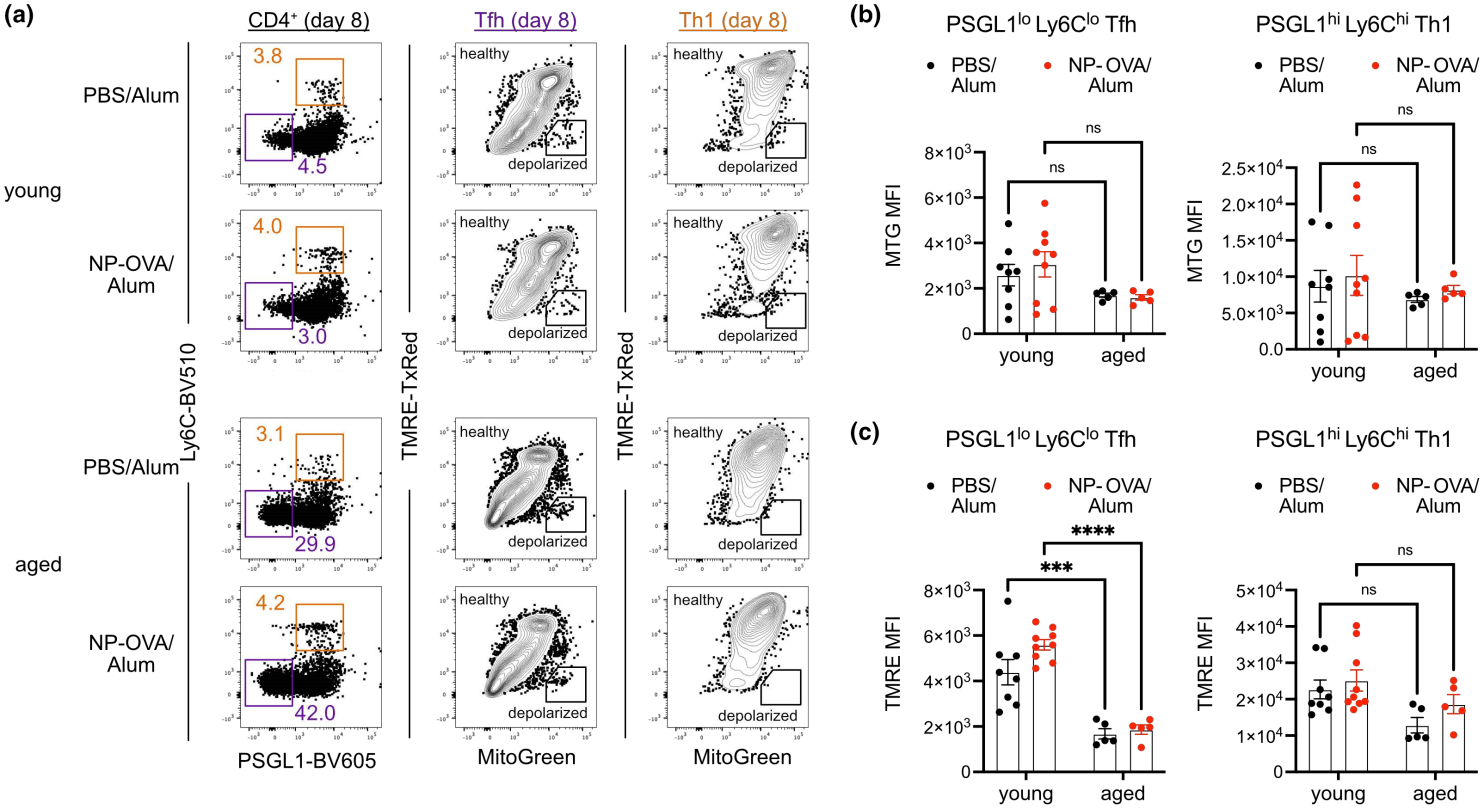
Aged Tfh cells exhibit signs of bioenergetic stress. (a) Young (2‐ to 3‐month‐old) and aged (18‐22‐month‐old) mice were immunized with NP‐OVA/Alum or PBS/Alum, and the differentiation of CD4^+^ T cells into Tfh (PSGL1^lo^Ly6C^lo^) or Th1 (PSGL1^hi^Ly6C^hi^) subsets was analyzed 8 days post‐immunization (dpi) with assessment of mitochondrial activity by MitoTracker Green (MTG) and TMRE‐TexasRed. (b) MTG mean fluorescence intensities (MFI) and (c) TMRE MFI within Tfh and Th1 of young and aged mice 8 dpi. Data compiled from two experiments with each data point representing a mouse, bars represent mean ± SEM. Tested by two‐way ANOVA with Sidak correction for multiple comparisons, *p*‐values: **** < 0.0001, *** < 0.001, ns: not significant.

Activation of naïve CD4^+^ T cells involves the rapid remodeling of metabolic programs to meet the demands of proliferation and differentiation. To realize their energy needs, CD4^+^ T cells shift from oxidative phosphorylation to aerobic glycolysis, thereby transitioning from catabolic to anabolic metabolism (Buck et al., 2015). However, in the case of Tfh cells, *Bcl6* induction inhibits glycolysis and engages catabolic programs, akin to the metabolic changes experienced by memory T cells (Oestreich et al., 2014). To determine whether regulation of the aerobic glycolysis could be altered in aged CD4^+^ T cells upon activation, we used previously defined surface markers, PSGL‐1^lo^Ly6C^lo^ for Tfh and PSGL1^hi^Ly6C^hi^ for Th1 (Ray et al., 2015), to compare mitochondrial readouts MitoTracker Green (MTG) and TMRETexasRed (Figure 2a) in splenic T cells from mice 8 dpi. Depolarized cells, labeled as MTG^hi^TMRE^lo^, were excluded from metabolic analysis (Figure 2a). PSGL‐1^lo^Ly6C^lo^ Tfh cells were increased in frequency with age, consistent with our quantification based on CXCR5, PD‐1, and ICOS, while frequencies of PSGL‐1^hi^Ly6C^hi^ Th1s were similar between the ages (Figure S3). Mitochondrial mass, as assessed by MTG labeling, trended toward a slight reduction in healthy (i.e., non‐depolarized) Tfh and Th1 cells from immunized aged mice, though this was not statistically significant (Figure 2b). Mitochondrial membrane potential, as measured by TMRE, was significantly decreased in healthy Tfhs but not Th1s from immunized aged mice when compared to immunized young mice (Figure 2c). Thus, our data indicates that the aging GC response consists of accumulated Tfh cells with decreased mitochondrial membrane potential, suggestive of bioenergetic stress.

**FIGURE 3:**
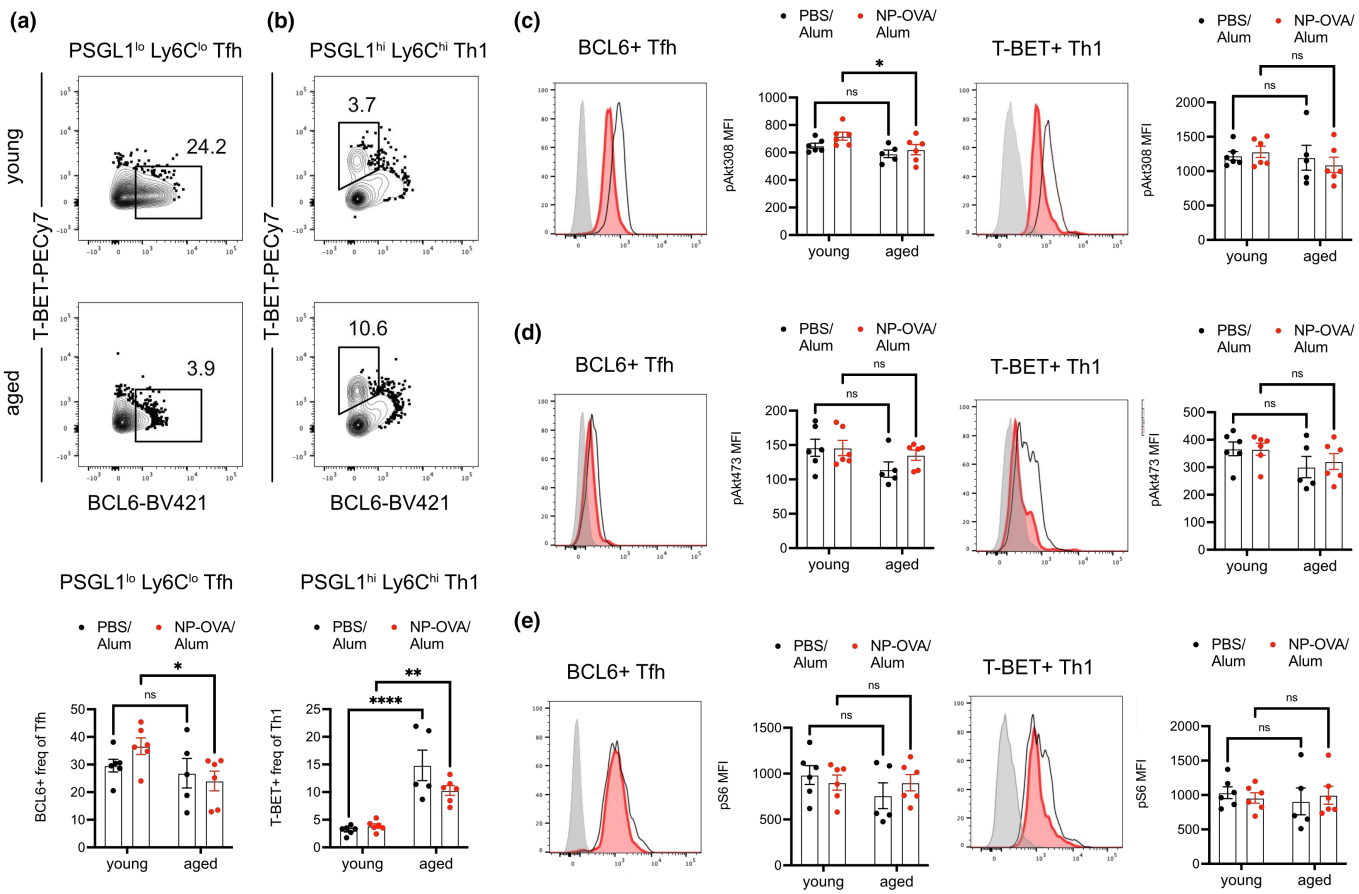
Intracellular signaling downstream of TCR is impaired in aged Tfh cells. Young (2‐3‐month‐old) and aged (18‐ to 22‐month‐old) mice were immunized with NP‐OVA/Alum or PBS/Alum, and the Tfh (PSGL1^lo^Ly6C^lo^) or Th1 (PSGL1^hi^Ly6C^hi^) subsets of CD4^+^ T cells were analyzed 8 days post‐immunization (dpi) for phosphorylated targets of the Akt–mTOR signaling pathway. (a) Frequency of PSGL1^lo^Ly6C^lo^ Tfh cells with upregulated BCL6 expression in young and aged mice 8 dpi. (b) Frequency of PSGL1^hi^Ly6C^hi^ Th1 cells with upregulated T‐BET expression in young and aged mice 8 dpi. Histograms and mean fluorescence intensities (MFI) of (c) pAkt308, (d) pAkt473, (e) pS6 within BCL6^+^ Tfh and T‐BET^+^ Th1 cells in young (blackoutlined curve) and aged (solid red curve) mice 8 dpi. AF647 channel FMO shown in gray. Data compiled from two experiments with each data point representing a mouse, bars represent mean ± SEM. Tested by two‐way ANOVA with Sidak correction for multiple comparisons, *p*‐values: **** < 0.0001, ** < 0.01, * < 0.05, ns: not significant.

Intracellular staining of the accumulated PSGL‐1^lo^Ly6C^lo^ Tfh in immunized aged mice confirmed that a reduced proportion of them had upregulated BCL6 expression (Figure 3a). This was unlike aged PSGL‐1^hi^Ly6C^hi^ Th1 cells, which were able to upregulate their master transcription factor T‐BET (Figure 3b). To further investigate the difference in aged Tfh differentiation, we stained for phosphorylated targets of the Akt–mTOR pathway, as this signaling axis plays an important role in Tfh differentiation (Ray et al., 2015). BCL6^+^ Tfh in immunized aged mice had reduced pAkt308, but not pAkt473 or pS6, when compared to those in young mice (Figure 3c–e). This is unlike the case of T‐BET^+^ Th1, which had similar expression levels of pAkt308, pAkt473, and pS6 between the ages (Figure 3c–e). Because of its interactions with the mTOR pathway, we also analyzed expression of phosphorylated AMPK, but found that it was similar between ages for both Tfh and Th1 (Figure S4). Thus, our in vivo studies in immunized mice reveal metabolic changes in early stages of Tfh differentiation that are altered in peptide‐immunized aged mice.

### 3.3 Aged CD4 ^+^ naïve T cells retain proliferative potential but cannot upregulate CD40L

**FIGURE 4:**
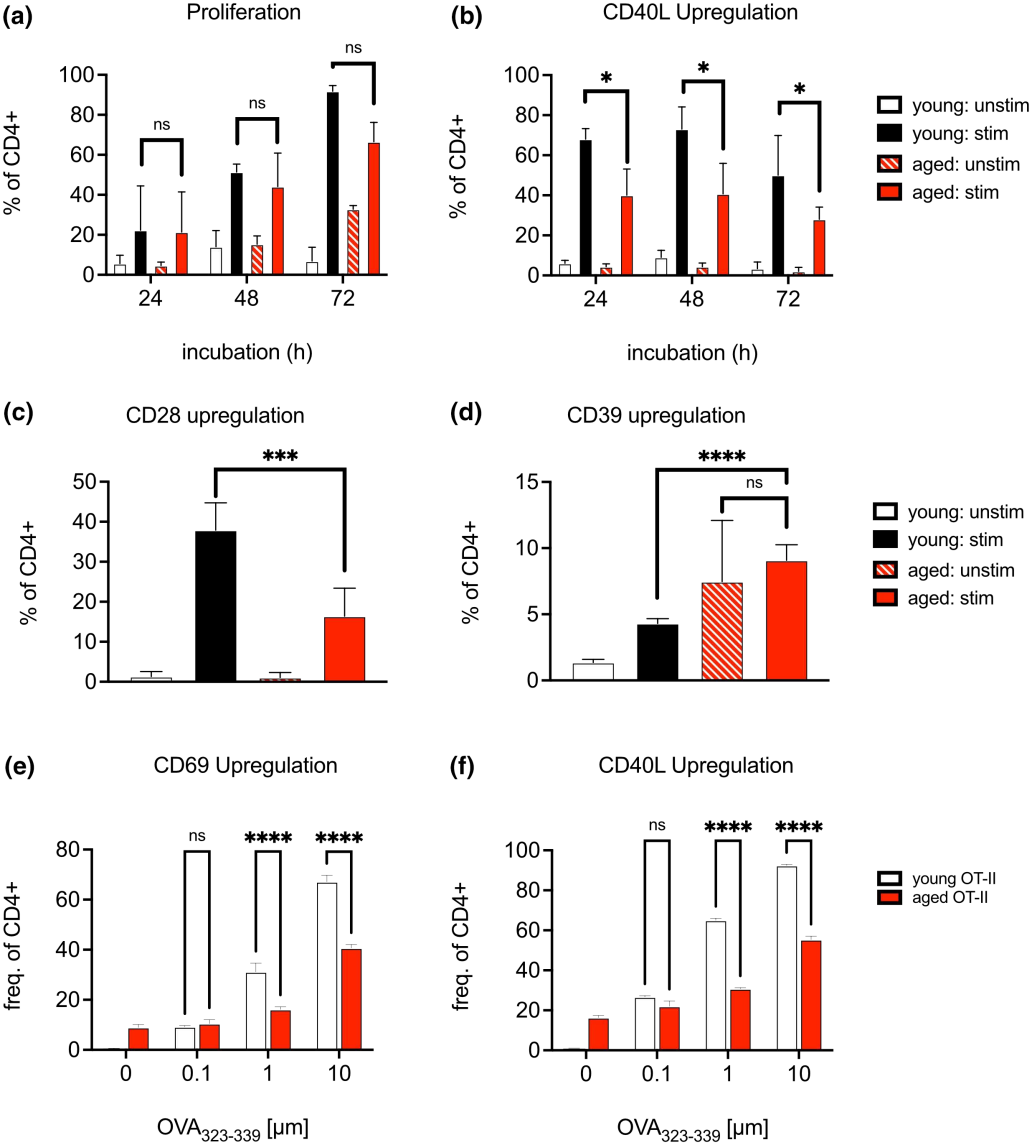
Aged CD4+ naïve T cells retain proliferative potential but cannot upregulate CD40L. (a) Frequencies of naive CD4^+^ T cells sourced from young (2‐3‐month‐old) and aged (16‐20‐month‐old) mice that have undergone proliferation and (b) CD40L upregulation in response to in vitro TCR crosslinking with anti‐CD3 and anti‐CD28 after various incubation periods. (c) Frequencies of naive CD4^+^ T cells sourced from young (2‐3‐month‐old) and aged (16‐20‐month old) mice that have mobilized surface CD28 and (d) express CD39 in response to in vitro TCR crosslinking with anti‐CD3 for 48 h. Frequencies of naïve CD4^+^ T cells sourced from young and middle‐aged (12‐month‐old) OT‐II mice that have undergone (e) CD69 upregulation and (f) CD40L upregulation in response to in vitro co‐culture for 48 h with young splenocytes prepulsed with various concentrations of OVAp_323‐339_. Data compiled from two to three experiments with the experimental means of triplicate wells, bars represent mean ± SEM. Tested by two‐way ANOVA with Sidak correction for multiple comparisons, *p*‐values: **** < 0.0001, *** < 0.001, * < 0.05, ns: not significant.

The defects in CD4^+^ T cell function observed could be certainly linked to cell‐intrinsic changes with age (Srinivasan et al., 2021), though it is not clear if this is due to age‐induced dysfunction of naïve CD4^+^ T cells, or driven predominantly by reduced proportions of naïve CD4^+^ T cells with age (Nikolich‐Žugich, 2014). To assess the potential for aged naïve CD4^+^ T cells to respond to TCR engagement, we isolated naïve CD4^+^ T cells from young and aged mice and stimulated them with plate‐bound antibodies against CD3 and CD28 for 72 h. Cellular proliferation, as measured by dilution of CellTrace Violet, was comparable between young and aged T cells, though the frequency of proliferating aged T cells was slightly decreased at the 72‐h time‐point (Figure 4a). We furthermore measured surface expression of CD40L, as this molecule plays a critical role in the stabilization of CD4^+^ T cell:B cell interactions during germinal center induction (Grewal & Flavell, 1998). Unlike early‐stage proliferative activity, the frequency of aged CD4^+^ T cells that upregulated CD40L was significantly decreased as compared to young T cells at all time points assessed (Figure 4b). To further investigate T cell intrinsic changes in TCR activation, we stimulated naïve CD4^+^ T cells isolated from young and aged mice with plate‐bound antibody against CD3 alone, and assessed the mobilization of CD28 after 48 h (Butler et al., 2002). Surface CD28 expression was significantly reduced in aged CD4^+^ T cells upon stimulation, as compared to young CD4^+^ T cells (Figure 4c). Stimulated aged CD4^+^ T cells also had increased induction of CD39 as compared to young CD4^+^ T cells (Figure 4d). Our studies determined that TCR cross‐linking in aged naïve T cells resulted in reduced CD40L and CD28 expression as compared to young cells, though proliferative capacity was maintained.

To determine whether activation of CD4^+^ naïve T cells by their cognate antigen was also impacted by age, we stimulated CD4^+^ naïve T cells isolated from OT‐II TCR transgenic mice, which express TCRs with high specificity to the OVA_323‐339_ peptide (OVAp_323‐339_) presented within the context of MHC‐II (Barnden et al., 1998). Though previous literature have used transgenic mice as a bulk source for aged naïve T cells (Eaton et al., 2004; Haynes et al., 1999), we still found that the relative proportions of naïve CD4^+^ T cell compartment was reduced in OT‐II mice as CD44^hi^CD62L^lo^CD4^+^ T cells expanded with age, despite being housed in specific pathogen free conditions (Figure S5A). We further observed that OT‐II mice were susceptible to splenomegaly with age, and thus we limited our aging of TCR transgenic animals to 12 months, or approximately middle‐aged (Flurkey et al., 2007), and discarded animals with splenomegaly or other indications of poor health. CD44^lo^CD62L^hi^ naïve CD4^+^ T cells isolated from young or middle‐aged OT‐II mice were co‐cultured with bulk splenocytes from young C57BL/6J mice pulsed varying concentrations of OVAp_323‐339_ for 48 h. At all peptide concentrations, naïve CD4^+^ T cells from middle‐aged OT‐II mice were diminished in their ability to upregulate CD44 (Figure S5B). Strength of TCR signaling was further determined by upregulation of CD69, and we found that middle‐aged OT‐II CD4^+^ T cells had reduced CD69 expression as compared to young cells when stimulated with 1–10 μM peptide concentrations (Figure 4e). As with TCR crosslinking, we found that middle‐aged OT‐II CD4^+^ T cells had decreased upregulation of CD40L when stimulated with 1–10 μM peptide concentrations (Figure 4f). Our studies indicate that while aging naïve CD4^+^ T cells retain much of their capacity to proliferate upon TCR ligation, they have reduced levels of CD69 and CD40L upregulation, which could weaken their support of B cell activation.

### 3.4 Aged APCs cannot promote effective expansion of CD4 ^+^ CD40L ^+^ T cells

**FIGURE 5:**
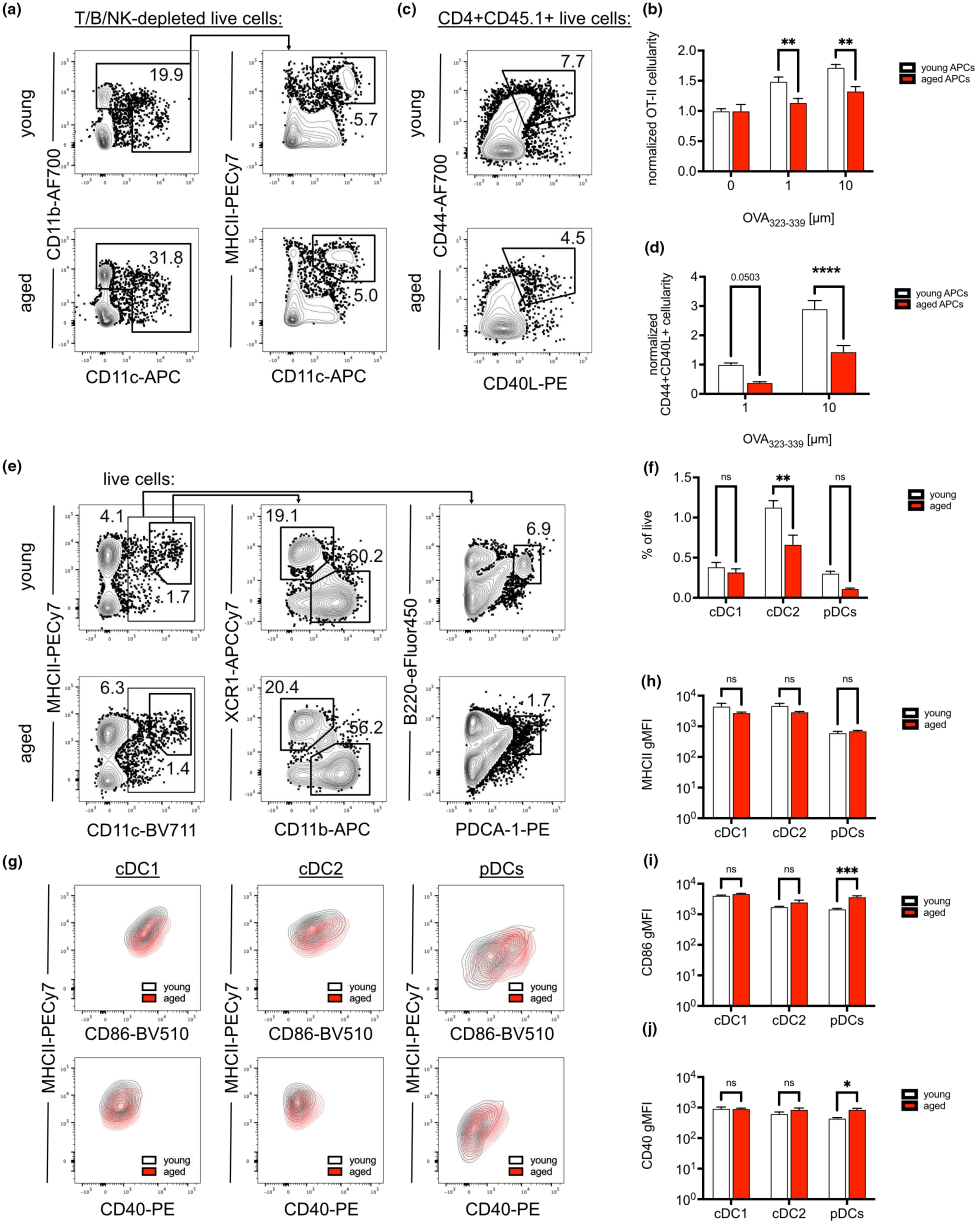
Aged APCs cannot promote effective expansion of CD4^+^CD40L^+^ T cells. (a) Characterization of young and middle‐aged mouse splenocytes after depletion of T, B, and NK cells. (b) Cellularity of OT‐II CD4^+^ T cells after in vitro co‐culture for 48 h with T/B/NKdepleted young and aged splenocytes pulsed with various concentrations of OVAp_323‐339_, normalized to respective cell numbers in unstimulated wells. (c) Upregulation of CD44 and CD40L by OT‐II CD4^+^ T cells after stimulation by T/B/NK‐depleted young and aged splenocytes pulsed with 1 μM OVAp_323‐339_. (d) Cellularity of OT‐II CD4^+^CD44^+^CD40L^+^ T cells after in vitro co‐culture for 48 h with T/B/NK‐depleted young and aged splenocytes pulsed with various concentrations of OVAp_323‐339_, normalized to cellularity in wells stimulated by young APCs pulsed with 1 μM peptide. (e) Spleens from young (2‐3‐month‐old) and aged (18‐20‐month‐old) mice were analyzed at steady state to characterize dendritic cell (DC) subsets. (f) Frequencies of conventional DC subsets 1 and 2 (cDC1 and cDC2) and plasmacytoid DCs (pDCs) within live cells in young and aged spleens. (g) Steady‐state expression of costimulatory molecules within young and aged splenic cDC1, cDC2, and pDCs. Geometric mean fluorescence intensities (gMFI) for expression of (h) MHCII, (i) CD86, and (j) CD40 within young and aged splenic cDC1, cDC2, and pDCs. Compiled from two experiments, tested by two‐way ANOVA with Sidak correction for multiple comparisons, *p*‐values: *** < 0.001, ** < 0.01, * < 0.05, ns: not significant.

Our in vitro studies indicated that the potential for CD40L induction was limited with T cell age, and we further questioned whether exposure to the aged tissue microenvironment could also have an effect. To explore this hypothesis, we co‐cultured naïve CD4^+^ T cells isolated from young OT‐II mice with bulk splenocytes from either young or middle‐aged (12‐month‐old) C57BL/6J mice pulsed with varying concentrations of OVAp_323‐339_ for 48 h. CD3^+^ T cells constituted comparable proportions of the spleens of the young and middle‐aged mice (Figure S6A) indicating that OT‐II T cells would encounter similar numbers of antigen‐presenting cells (APCs) within the bulk splenocytes, though the APC subset composition would be specific to the age of the mice. OT‐II cell numbers in response to the different antigen concentrations remained comparable between the two APC‐age conditions (Figure S6B). Strength of TCR signaling, as determined by CD69 expression, remained similar between APC ages, though T cells stimulated by aged APCs pulsed with the intermediate dose of 1 μM OVAp_323‐339_ had slightly, though not significantly, decreased CD69 expression (Figure S6B). Induction of CD40L and CD39 was also similar when OT‐II T cells were co‐cultured with young versus middle‐aged APCs (Figure S6B), though we noted a trend for aged APCs to induce slightly less CD40L and more CD39. To better ascertain the influence of aged APCs, we conducted assays in which T cells, B cells, and NK cells had been depleted from young and aged splenocytes (Figure 5a) and pulsed with 1 or 10 μM of OVAp_323_339 peptide before co‐culture with CD4^+^ naïve T cells from young OT‐II mice. Here, T/B/NK‐depleted APCs were plated at 2:1 ratio with T cells, versus 5:1 ratio as conducted in experiments with bulk splenocytes. While young and aged T/B/NK‐depleted APCs were able to induce similar frequencies of CD44, CD40L, and CD69 expression (Figure S6C), greater expansion of young OT‐II CD4^+^ T cells was supported by young APCs (Figure 5b), translating into greater numbers of OT‐II CD4^+^ CD44^+^CD40L^+^ T cells (Figure 5c,d). Thus, while aged APCs maintain the ability to activate naïve CD4^+^ T cells, the magnitude of CD4^+^ T cell expansion is limited, which may constrain the pool of activated CD4^+^ T cells.

We further characterized the dendritic cell (DC) compartments within young (2‐ to 3‐month‐old) and aged (18‐22‐month‐old) C57BL/6J spleens, which include 2 subsets of conventional DCs and plasmacytoid dendritic cells (pDCs) (Figure 5e). Proportions of XCR1^+^ cDC1 and pDCs were comparable between young and aged spleens at steady state, whereas the frequency of CD11b^+^ cDC2 was significantly reduced in aged mice (Figure 5f). All subsets trended toward slightly, though not significantly, reduced cell numbers within the aged spleen (Figure S6D). Characterization of MHCII and costimulatory molecule expression did not reveal obvious differences at steady state, except for increased expression of CD86 and CD40 among aged pDCs (Figure 5g–j). We did not observe obvious differences in transcript expression of cytokines *Il6*, *Il21*, and *Tgfb1* between young and aged spleens at steady state, though notably, expression of *Baff* was consistently elevated (Figure S6E). Thus, we found that the splenic DC compartment of aged mice was broadly similar to that in young mice.

### 3.5 Antigen‐specific germinal center activation is impaired in the aged microenvironment

**FIGURE 6:**
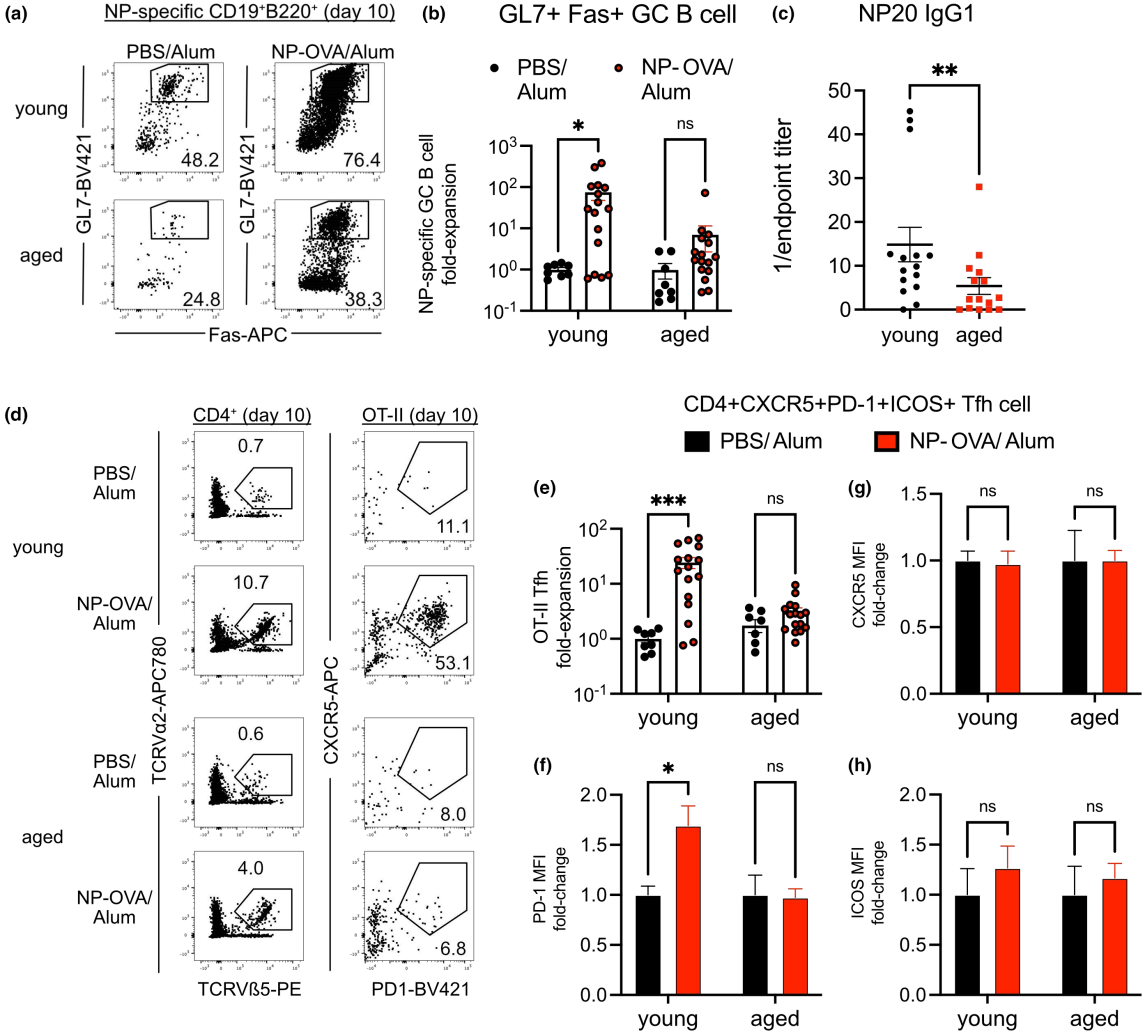
Antigen‐specific germinal center activation is impaired in the aged microenvironment. (a) OT‐II CD4^+^ naïve T cells and B^1‐8^ B cells, isolated from young (2–3 month‐old) mice, were transferred into young and aged (18‐ to 22‐month‐old) mice prior to immunization with NP‐OVA/Alum or PBS/Alum, and the differentiation of NP‐specific GL7^+^Fas^+^ GC B cells was analyzed 10 days post‐immunization (dpi). (b) Fold‐expansion of GL7^+^Fas^+^ GC B cells within NP‐specific B cells, normalized to young mice treated with PBS/Alum, was analyzed 10 dpi. (c) Endpoint titers of NP20‐specific IgG1 antibodies from sera of immunized young and aged mice 10 dpi. (d) Differentiation of OT‐II CD4^+^ T cells into Tfh phenotype within young and aged mice 10 dpi. (e) Fold‐expansion of CD4^+^CXCR5^+^PD‐1^+^ICOS^+^ Tfh cells within OT‐II cells, normalized to young mice treated with PBS/Alum, was analyzed 10 dpi. Fold‐change of mean fluorescence intensities (MFI) of (f) PD‐1, (g) CXCR5, and (h) ICOS of OT‐II Tfh within young and aged mice 10 dpi. Data compiled from four experiments with each data point representing a mouse, bars represent mean ± SEM. Tested by two‐way ANOVA with Sidak correction for multiple comparisons, *p*‐values: *** < 0.001, ** < 0.01, * < 0.05, ns: not significant.

To investigate whether the aged microenvironment could impact germinal center expansion, we adoptively transferred CD4^+^ naïve T cells, isolated from young OT‐II TCR transgenic mice, and B cells, isolated from young B^1‐8^; Jκ^−/−^ transgenic mice, whose B cells are specific for NP (Sonoda et al., 1997), into young and aged (18‐ to 22‐month‐old) hosts 24 h before intraperitoneal immunization with NP‐OVA in alum. Analyses of splenic B cells 10 dpi indicated that expansion of NP‐specific GC B cells was impaired in immunized aged hosts (Figure 6a,b), which corresponded to reduced recovery of NP‐specific IgG1 from aged mouse serum (Figure 6c).

Young donor‐derived, OT‐II CD4^+^ T cells within the aged hosts were also limited in their ability to expand and differentiate into the Tfh phenotype as compared to those transferred into young mice (Figure 6d,e). Comparison of Tfh‐specific surface markers revealed that OT‐II Tfh cells had reduced upregulation of PD‐1 signal when activated within the aged host (Figure 6f), whereas there were no significant differences in the expression levels of CXCR5 (Figure 6g) and ICOS (Figure 6h). These experiments suggest that the efficacy of GC activation is influenced by the aged tissue microenvironment, even when lymphocytes derived from young organisms are activated against their high‐affinity antigen.

### 3.6 CD4 ^+^ T cells activated in the aged microenvironment have reduced BCL6 induction

**FIGURE 7:**
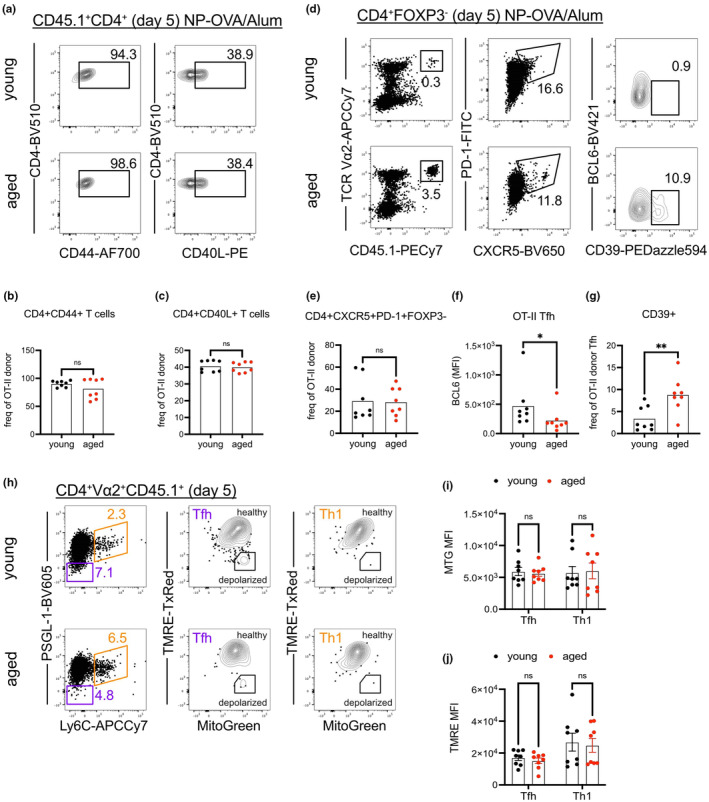
CD4^+^ T cells activated in the aged microenvironment have reduced BCL6 induction. OT‐II CD4^+^ naïve T cells and B^1‐8^ B cells, isolated from young (2‐3‐month‐old) mice, were transferred into young and aged (18‐22‐month‐old) mice prior to immunization with NPOVA/Alum or PBS/Alum, and the expression of CXCR5, PD‐1, BCL6, CD39, and CD40L on OT‐II cells was analyzed 5 days post‐immunization (dpi). (a) Adoptively‐transferred OTII; CD45.1^+^ CD4^+^ T cells gated for CD44 and CD40L expression. Frequencies of OT‐II donor cells with (b) CD44 and (c) CD40L expression in young and aged mice 5 dpi. (d) Adoptively‐transferred OT‐II; CD45.1 CD4^+^CXCR5^+^PD‐1^+^ T cells gated for BCL6 and CD39 expression. CD4^+^CXCR5^+^PD‐1^+^FOXP3^−^ Tfh phenotype within young and aged mice 5 dpi. (e) Frequencies of OT‐II donor cells with CXCR5^+^PD‐1^+^ expression in young and aged mice 5 dpi. (f) BCL6 mean fluorescence intensities (MFI) on OT‐II Tfh within young and aged mice 5 dpi. (g) Frequencies of CD39^+^ cells of OT‐II Tfh within young and aged mice 5 dpi. (h) Differentiation of donor OT‐II CD4^+^ T cells into Tfh (PSGL1^lo^Ly6C^lo^) or Th1 (PSGL1^hi^Ly6C^hi^) subsets was analyzed 5 days post‐immunization (dpi) with assessment of mitochondrial activity by MitoTracker Green (MTG) and TMRE. (i) MTG and (j) TMRE MFI of donor OT‐II Tfh and Th1 cells within young and aged mice 5 dpi. Data compiled from two to four experiments with each data point representing a mouse, bars represent means ± SEM. Tested by two‐way ANOVA with Sidak correction for multiple comparisons, *p*‐values: ** < 0.01, * < 0.05, ns: not significant.

To further define impaired Tfh differentiation in the aged microenvironment, we analyzed young, adoptively transferred OT‐II CD4^+^ T cells at an earlier time point, 5 dpi. We found similar frequencies of CD44 and CD40L induction among donor OT‐II CD4^+^ T cells transferred into young and aged hosts (Figure 7a–c). Because of the reduced overall proportions of CD4^+^ T cells in aged mice (Figure S2A), adoptively transferred OT‐II cells comprised a larger proportion of CD4^+^ T cells in immunized aged mice (Figure 7d, first panels). Regardless, the frequency of transferred OT‐II cells that adopted the CXCR5^+^PD‐1^+^ phenotype was not significantly different between immunized young or aged hosts at 5 dpi (Figure 7e). Despite adopting similar frequencies of the CXCR5^+^PD‐1^+^ phenotype, young OT‐II CXCR5^+^PD‐1^+^ Tfh cells within immunized aged mice had reduced BCL6 expression (Figure 7f), and increased expression of CD39 (Figure 7g). We furthermore analyzed fluorescence readouts of mitochondrial function, as conducted for immunized polyclonal aged CD4^+^ T cells in Figure 3, for the transferred young OT‐II cells at 5 dpi (Figure 7h). Subsetting based on Ly6C and PSGL1 expression also indicated that similar proportions of donor cells adopted Th1 or Tfh fates regardless of the host age (Figure S7A). Frequencies of depolarized OT‐II cells were comparable between young and aged hosts (Figure S7B). MitoTracker Green readout for mitochondrial mass indicated that Tfh and Th1 cells were not impacted by host age (Figure 7i). Mitochondrial membrane potentials, as measured by TMRE staining, were also comparable whether OT‐II Tfh and Th1 cells were activated within young or aged hosts (Figure 7j). Thus, our data reveal that even short‐term exposure to the aged tissue microenvironment can impair some, but not all, aspects of the differentiation program for young, antigen‐specific CD4^+^ T cells upon immunization.

## 4 DISCUSSION

Improved health outcomes against infectious disease for aging populations is a significant public health priority (Goronzy & Weyand, 2013). Even with this interest and with recent technological advances for characterizing biological age, investigation into the age‐related changes to the immune system has been challenging. For one, study of the immunization or vaccination response in situ requires the manipulation of the lymphoid tissues, which are difficult to sample from patients, especially within the time scale of immune activation. Additionally, as aging introduces changes at multiple levels of biological organization, at the molecular, cellular, and tissue level (Kennedy et al., 2014), it can be daunting to dissect its impact on a complex biological process such as the GC reaction. In our investigation, we have used the aged mouse model, which has been well‐appreciated for the similar structure of its immune system (Haley, 2003), in order to probe the aged lymphoid tissue microenvironment as it responds to immunization. Here, we focus on defining markers of CD4^+^ T‐cell activation and differentiation that are impaired in a cell‐intrinsic and/or ‐extrinsic fashion with age. Identification of murine factors within the GC response that are significantly impacted by aging will guide subsequent study of the immunization response within older humans.

Single‐cell RNA‐sequencing has confirmed that aged mice have a decreased naïve population that is offset by increased representation of CD4^+^ T cells with exhausted or regulatory signatures (Elyahu et al., 2019). In agreement with this and other broad CD4^+^ T cell subset profiling, we found that aged mice had increased proportions and numbers of CD4^+^ T cells that express PD‐1 and FOXP3. As others have recently shown, we also found that aging was associated with increased proportions and numbers of Tfh and Tfr (Almanan et al., 2020; Sage et al., 2015). While BCL6 expression was comparable between young and aged Tfh at steady‐state, as others have reported (Fang et al., 2016; Sage et al., 2015), upon immunization we found that aged Tfh had reduced levels of BCL6 as compared to young Tfh. This coincided with increased numbers of aged Tfh cells expressing CD39, whose activity is known to negatively regulate *Bcl6* (Cao et al., 2020; Srinivasan et al., 2021). CD39, along with CD73, have been evaluated in the context of chronic infection and cancer, as they promote immunosuppression in response to environmental inflammatory cues (Balança et al., 2021). Upregulation of inflammatory signaling pathways and increased baseline serum concentrations of inflammatory cytokines are well‐established hallmarks of aging (Bektas et al., 2017; Franceschi et al., 2018), and thus the aging milieu may promote the accumulation of Tfh‐like cells with reduced capacities to support GC responses and rather play immunosuppressive functions.

Like earlier studies (Haynes et al., 1999), we find that the proliferation of CD4^+^ T cells from aged mice in response to TCR crosslinking remains comparable to that of young mice within the first 72 h. Despite maintaining proliferative capacity, aged naïve CD4^+^ T cells had reduced upregulation of CD40L expression, also in agreement with previous results using bulk CD4^+^ T cells (Eaton et al., 2004; Yu et al., 2012). Further indication that TCR signaling may be altered in aged mice was reduced mobilization of the CD28 co‐receptor upon in vitro stimulation. Aged human CD4^+^ T cells have been characterized by an emergence of a long‐lived CD28^−^ population, which retained production of interferon‐κ and interleukin‐2, but lacked CD40L expression and were poor stimulators of B cells (Weyand et al., 1998). Mirroring our in vivo studies with immunized aged mice, aged CD4^+^ T cells had greater CD39 expression. Whereas others have shown that CD4^+^ T cells as a population have some functional impairments (Srinivasan et al., 2021), here we show in mice that aged naïve CD4^+^ T cells also bear defects in signaling downstream of TCR stimulation that would limit Tfh functions.

Given its importance to the GC response, the observed reduction in aged CD4^+^ T cell expression of CD40L identifies one vulnerability in the aged immune system (Linterman, 2014). Defective upregulation of CD40L and CD69 was observed when TCR transgenic CD4^+^ naïve T cells from middle‐aged mice were activated against their cognate ligand. This suggests that despite maintaining their capacity to proliferate when activated non‐specifically, aged CD4^+^ T cells experienced reduced antigen‐specific TCR activation. Transcriptional control of CD40L is regulated by intracellular pathways including NFκB and NFAT (Pham et al., 2005; Srahna et al., 2001), but, unlike CD69, not AP‐1 (Castellanos et al., 1997). Intrinsic changes to NFκB signaling has already been implicated for aging T cells (Bektas et al., 2014). In vivo, we had observed a very slight but significant reduction in pAkt308 in Tfh‐differentiated CD4^+^ T cells in immunized aged mice as compared to young mice, but not pAkt473, a difference which was not observed in Th1‐differentiated cells. While a thorough investigation of mammalian target of rapamycin (mTOR) signaling was beyond the scope of the current study, others have indicated that aging has differential effects on phosphorylation of Akt308 versus Akt473 and the subsequent engagement of mTOR complexes 1 and 2 within CD4^+^ T cells (Perkey et al., 2013).

Aged CD4^+^ T cells exhibited reduced CD40L upregulation, yet were able to maintain proliferative capacity in vitro. This phenotype was distinct from young naïve CD4^+^ T cells activated in vitro by APCs isolated from middle‐aged mice: here, the ability to elicit TCR activation and CD40L upregulation was only slightly diminished, and rather the expansion of activated CD4^+^ T cells was inhibited, thereby reducing the overall numbers of CD40L^+^ cells generated from the starting CD4^+^ T cell pool. This effect was more apparent when APCs in vitro were in limited ratios with T cells. Thus, aged CD4^+^ T cells in the aged tissue microenvironment may experience both effects, in which their ability to upregulate CD40L is impaired by intrinsic defects downstream of TCR stimulation, and the overall expansion of antigen‐specific T cells is reduced by the limitations of aged APCs. Characterization of splenic DCs from aged mice did not reveal any broad changes to their composition, other than a reduction in the frequency of cDC2, as others have shown (Stebegg et al., 2020). TCR ligation may be impacted by the efficacy of engagement by CD80 and CD86 on APCs with the costimulatory receptor CD28 on T cells, which modulate NFκB via PI3‐kinase (Schmitz & Krappmann, 2006). Though we found no change to steady state levels of costimulatory molecules on aged DCs, others have reported that aged murine DCs have reduced upregulation of CD86 in response to infection (Li et al., 2012). Others have shown that defective cDC2 activation could be rescued by treatment with a Toll‐like receptor‐7 agonist, though numbers of GC B cells were only modestly increased (Stebegg et al., 2020). Thus, inefficient T cell priming by aged DCs may be limiting the input of activated CD4^+^ T cells, just one of many factors that impair the overall magnitude of the aged GC response.

Unlike previous work (Eaton et al., 2004), we found that adoptively transferred naïve CD4^+^ T cells, sourced from young mice, were unable to restore antigen‐specific GC B cell induction within aged hosts. This occurred despite the co‐transfer of young, antigen‐specific naïve B cells, which robustly expanded when transferred to young hosts. The transferred CD4^+^ T cells failed to expand to the same extent as they did within the young hosts, and furthermore had reduced induction of certain Tfh‐specific markers, including PD‐1 and BCL6. TCR engagement, in cooperation with ICOS and CXCR5, elicits the PI3‐kinase‐dependent signaling cascade that is required for T cell motility and subsequent Tfh recruitment (Xu et al., 2013). PD‐1, rapidly expressed upon activation (within 2 days in OT‐II cells) (Kitano et al., 2011), serves to concentrate Tfh to the GC, paradoxically by antagonizing ICOS and dampening CXCR3‐mediated chemotaxis (Shi et al., 2018). Like CD40L, discussed above, transcription of PD‐1 is regulated by NFAT (Dong et al., 2016), and thus reduced PD‐1 expression may be a result of impaired costimulation during TCR signaling. Defective PD‐1 induction further emphasizes the need to investigate how the aged microenvironment impairs CD4^+^ T cell intracellular signaling via NFAT and/or NFκB. Previous work had demonstrated that transferred OT‐II cells fail to accumulate in the GCs of immunized aged mice (Lefebvre et al., 2012), and our studies further suggest that dysregulated expression of PD‐1 could disturb the organization of the GC. More recently, it was demonstrated that Tfh cells have aberrant expression of CXCR4, which repositions them to the dark zone of the GC and impairs differentiation of follicular dendritic cells, contributing to diminished GC B cell expansion (Silva‐Cayetano et al., 2023). Age‐associated changes to lymph node stromal architecture has been described (Lancaster, 2023), and our previous work, using live‐cell 2‐photon imaging, found that naïve T cell motility was impaired within regions of accumulated fibrosis within the aged lymph nodes (Kwok et al., 2022). We hypothesize that our observations of decreased PD‐1 and CD40L are also indicative of imprecise spatial positioning of Tfh, which diminishes the efficiency of GC B cell interactions within the aged tissue microenvironment.

Previous work with OT‐II mice revealed that *Bcl6* expression also occurs rapidly upon CD4^+^ T‐cell activation (within 3 days of immunization), prior to Tfh clustering in the GCs (Kitano et al., 2011). Continued BCL6 expression is critical to the establishment of the GC, as BCL6 expression is required for long‐lasting B cell:T cell interactions within the follicle and for efficient surface expression CD40L (Liu et al., 2021). The *Bcl6* transcriptional program occurs in a STAT3‐dependent manner, downstream of IL‐21 and IL‐6 signaling in mice (Spolski & Leonard, 2014). Interestingly, aged Tfh from both mice and humans retain the ability to produce IL‐21 (Almanan et al., 2020; Zhou et al., 2014) and signal via STAT3 (Webb et al., 2021), and increased IL‐6 has been an established hallmark of aging (Bauernfeind et al., 2016), suggesting adequate cytokine availability with age, though the cytokine environment should be further investigated using high‐dimensional techniques. One prominent effect we measured was the expression of the ectonucleotidase CD39 in Tfh, which is known to inhibit the *Bcl6* program (Cao et al., 2020) but is highly expressed in aged human and murine CD4^+^ T cells (Cao et al., 2020; Fang et al., 2016). Aged CD39^+^CD4^+^ T cells have been shown to express higher levels of the transcription factor *Tbet* (Fang et al., 2016), which antagonizes PD‐1 expression and the Tfh fate (Xie et al., 2017). We found that young CD4^+^ T cells also had an increased tendency to differentiate into CD39^+^ cells within the aged microenvironment. Thus, we suspect that differentiating CD4^+^ T cells are faced with mixed cues from cytokines, damage‐associated molecular patterns, and other aging signals, which prevent full commitment to a functional Tfh fate.

Mitochondrial function regulates many aspects of CD4^+^ T cell differentiation and homeostasis. Upon activation, CD4^+^ T cells increase their bioenergetic metabolic pathways, which sustains their initial proliferative burst and cytokine production. Investigation into the mitochondrial metabolism of aged T cells has been limited, but some have indicated that metabolic rewiring of activated aged CD4^+^ T cells is defective (Ron‐Harel et al., 2018). We have expanded on these studies by comparing mitochondrial readouts between Tfh‐ and Th1‐differentiated CD4^+^ T cells in aged mice. While there was a trend toward decreased activity for Th1 cells, there was a clear reduction in the mitochondrial membrane potential of the accumulated, Tfh‐like cells in aged mice; further investigation is thus warranted as to whether these cells may be senescent (Passos et al., 2007). Even within the polyclonal population, we saw a tendency for immunization to increase the mean mitochondrial membrane potential of Tfh in young mice, but this effect was not observed with immunization in aged mice. In comparison, when sourced from young mice and adoptively transferred into aged recipients, antigen‐specific CD4^+^ T cells differentiated into Tfh or Th1 with comparable mitochondrial membrane activity as to those transferred into young hosts. This suggests that metabolic activity associated with CD4^+^ T‐cell activation is associated with cell‐intrinsic capabilities, and that defects we observed in PD‐1 and BCL6 are uncoupled from the metabolic program. Further work is needed to elucidate the cell‐extrinsic mechanisms by which CD4^+^ Tfh cell differentiation is impaired in the aged context.

In conclusion, our work demonstrates that aging contributes to cell‐intrinsic activation defects of naïve CD4^+^ T cells, and that aged‐associated defects to GC responses can be elicited by exposure to the aged tissue microenvironment. Our work supports a model in which the aged microenvironment provides cues that drive CD39 expression of Tfh‐fated cells, which is associated with impaired induction of BCL6. Without full commitment, expression of PD‐1 is also impaired, which provides further pressure against the *Bcl6* program. The expansion of CD40L‐expressing CD4^+^ T cells is constrained within the aged context, likely contributing to limited availability of T cell help of B cells. Future work must determine the mechanisms by which BCL6, PD‐1, and CD40L are impaired by the aged microenvironment, and how these changes impair Tfh support of GC B cell differentiation.

## AUTHOR CONTRIBUTIONS

J.L. devised experiments, J.F., I.A‐B., and N.K. performed experiments, J.F., I.A‐B., N.K., and J.L. analyzed data, and J.F. and J.L. wrote the manuscript.

## FUNDING INFORMATION

Research was supported by NIH/NIA R01 AG080037 and the Mayo Clinic Kogod Center on Aging Innovation Award UL1R002377 to J.L. I.A‐B. is a recipient of a FPU fellowship (FPU18/05752) from the Spanish Ministry of Science and a Company of Biologists Short Travelling Fellowship.

## CONFLICT OF INTEREST STATEMENT

The authors have no conflicts of interest to disclose.

## Supporting information


Appendix S1:

## Data Availability

Primary data and research tools used in this study are available from the corresponding author upon reasonable request.
