# Supplementary material for: The aged microenvironment impairs BCL6 and CD40L induction in CD4 + T follicular helper cell differentiation
Source: Aging Cell. 2024 Mar 13;23(6):e14140. doi: 10.1111/acel.14140 (PMC11296098; doi:10.1111/acel.14140)
Supplement: Supplementary file 1 — Appendix S1: [file ACEL-23-e14140-s001.pdf]

The aged microenvironment impairs BCL6 and CD40L induction in $CD4^+$ T follicular helper cell differentiation

Jacob S. Fisher, Irene Adán-Barrientos, Naveen R. Kumar, and Jessica N. Lancaster

Supplementary Information:

Supplementary Figures 1-7

Table 1

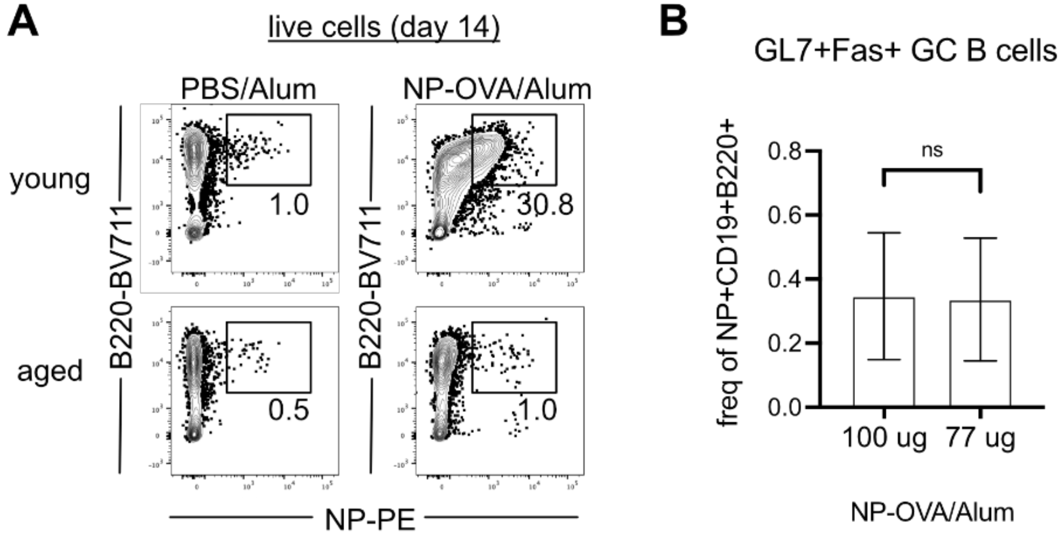

Supplementary Figure 1, in support of Figure 1. A. Young (2-3-month-old) and aged (18-22-month-old) mice were immunized with NP-OVA/Alum or PBS/Alum, and the differentiation of GC B cells was analyzed 14 days post-immunization (dpi). Gating on NP-binding B220^{+} lymphocytes within the spleens of young and aged mice 14 dpi. **B.** Young mice were immunized with 100 micrograms or 77 micrograms of NP-OVA/Alum, and the frequencies of GL7^{+}Fas^{+} GC B cells within NP-specific B220^{+}CD19^{+} B cells were analyzed 14 dpi.

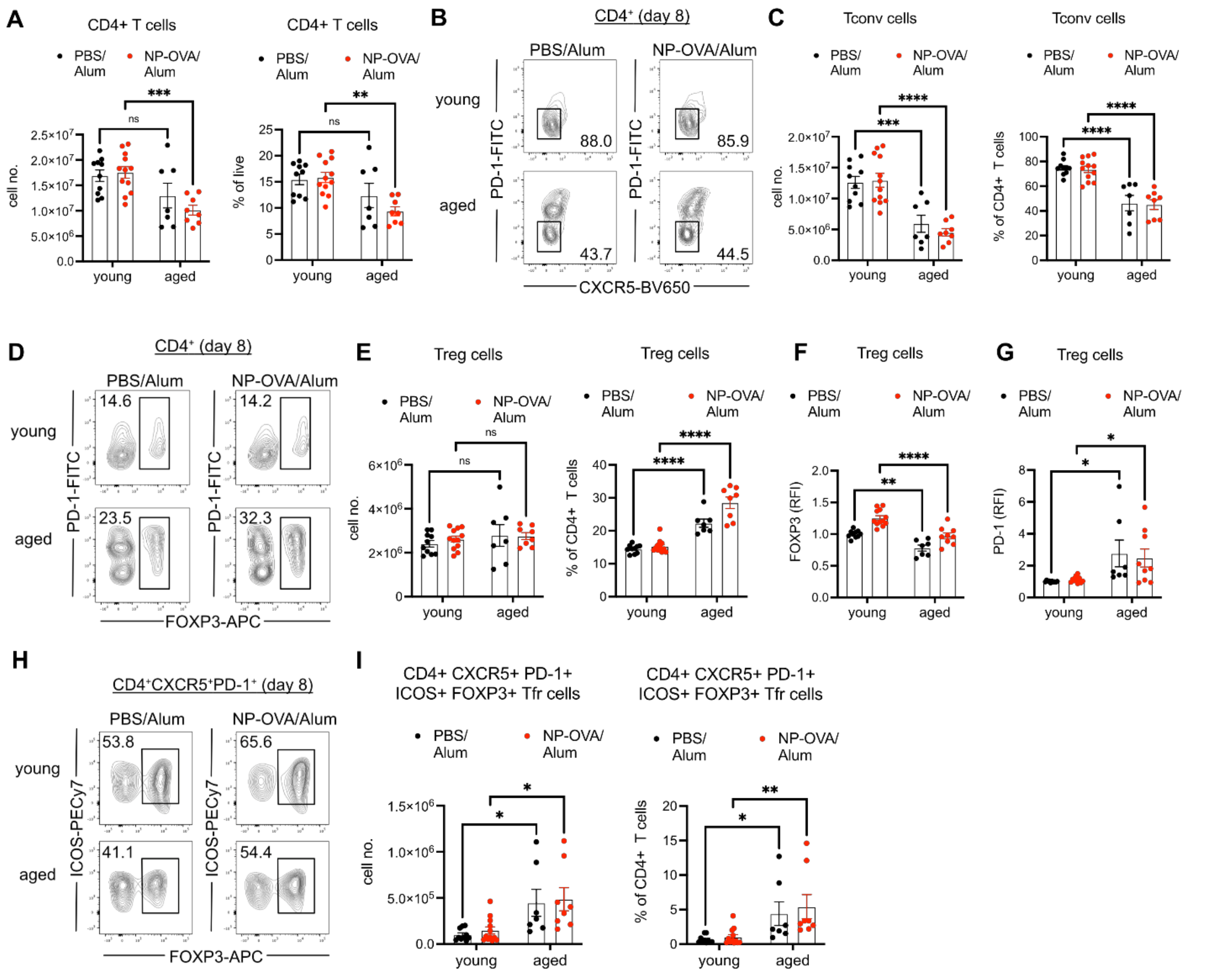

Supplementary Figure 2, in support of Figure 1. Young (2-3-month-old) and aged (18-22-month-old) mice were immunized with NP-OVA/Alum or PBS/Alum, and the differentiation of Tfh cells was analyzed 8 days post-immunization (dpi). **A.** Total CD4$^+$ T cell numbers and their frequencies within the spleens of young and aged mice 8 dpi. **B.** PD-1$^-$CXCR5$^-$ conventional T cells (Tconv) within CD4$^+$ T cells of young and aged mice 8 dpi. **C.** Total CD4$^+$ Tconv cell numbers and their frequencies within CD4$^+$ T cells of young and aged mice 8 dpi. **D.** FOXP3$^+$ regulatory T cells (Treg) within CD4$^+$ T cells of young and aged mice 8 dpi. **E.** Total CD4$^+$ Treg cell numbers and their frequencies within CD4$^+$ T cells of young and aged mice 8 dpi. Relative fluorescence intensities (RFI) of **F.** FOXP3 and **G.** PD-1 on CD4$^+$ Treg of young and aged mice 8 dpi, normalized to young mice treated with PBS/Alum. **H.** FOXP3$^+$ T follicular regulatory cells (Tfr) within CD4$^+$CXCR5$^+$PD-1$^+$ Tfh cells of young and aged mice 8 dpi. **I.** Total CD4$^+$CXCR5$^+$PD-1$^+$ICOS$^+$FOXP3$^+$ Tfr cells and their frequencies within CD4$^+$ T cells of young

and aged mice 8 dpi. Data compiled from 3 experiments with each data point representing a mouse, bars represent means ± SEM. Tested by two-way ANOVA with Sidak correction for multiple comparisons, *p*-values: **** < 0.001, *** < 0.005, ** < 0.01, * < 0.05, ns: not significant.

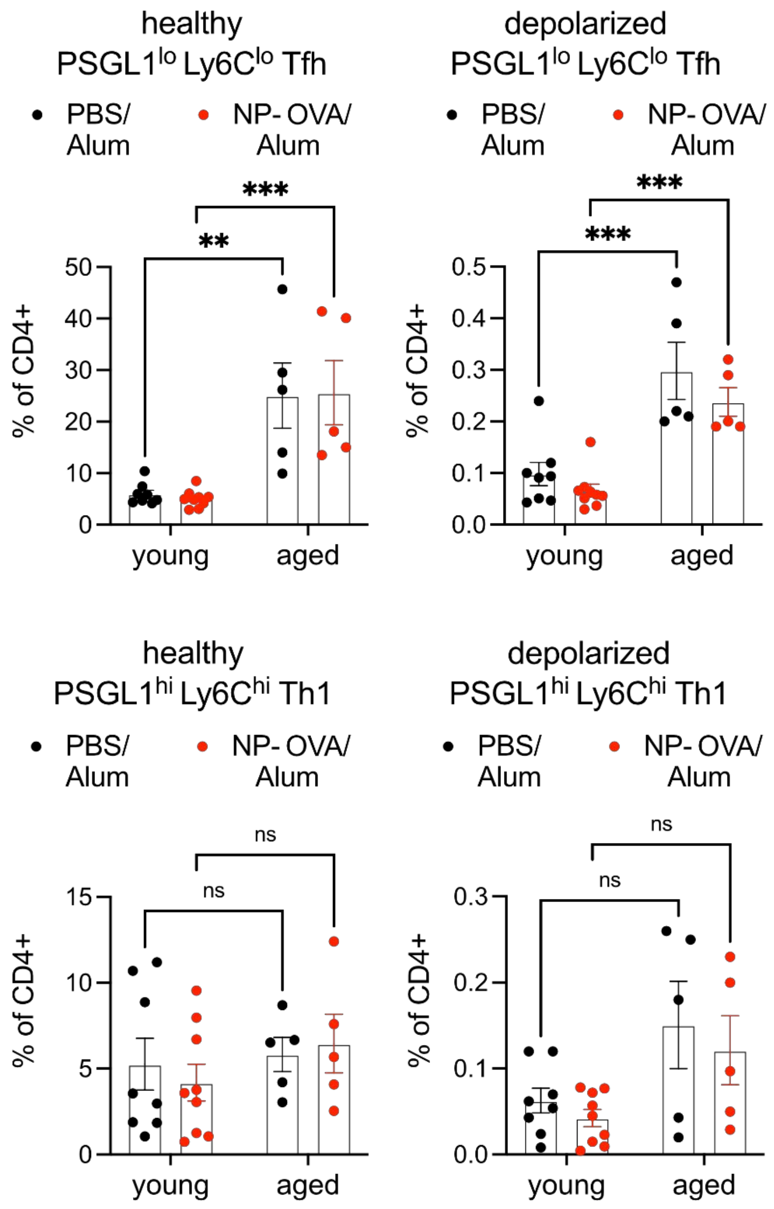

Supplementary Figure 3, in support of Figure 2. Young (2-3-month-old) and aged (18-22-month-old) mice were immunized with NP-OVA/Alum or PBS/Alum and analyzed 8 days post-immunization (dpi). Frequencies of Tfh and Th1 within $CD4^+$ T cells based on gating for scheme using markers PSGL-1 and Ly6C and depolarized cells based on gating Tfh and Th1 subsets by TMRE-$Red^{hi}MTG^{lo}$. Data compiled from 3 experiments with each data point representing a mouse, bars represent means ± SEM. Tested by two-way ANOVA with Sidak correction for multiple comparisons, *p*-values: *** < 0.005, ** < 0.01, ns: not significant.

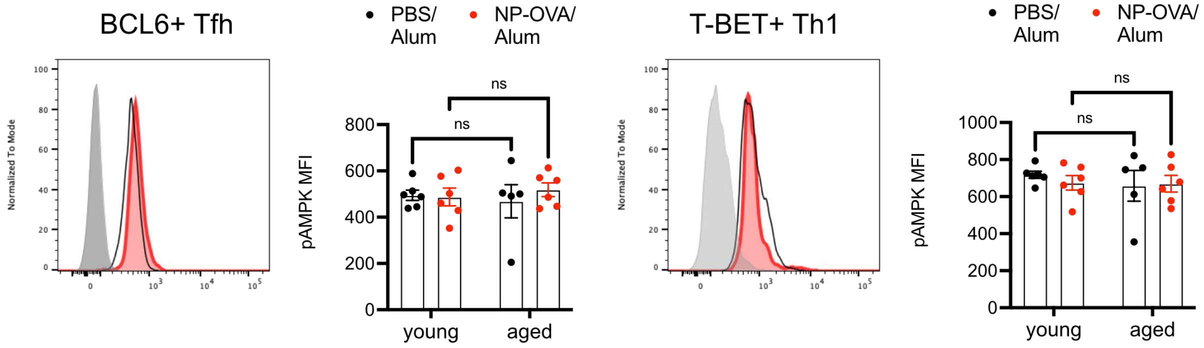

Supplementary Figure 4, in support of Figure 3. Young (2-3-month-old) and aged (18-22-month-old) mice were immunized with NP-OVA/Alum or PBS/Alum, and the Tfh (PSGL1^{lo}Ly6C^{lo}) or Th1 (PSGL1^{hi}Ly6C^{hi}) subsets of CD4^{+} T cells were analyzed 8 days post-immunization (dpi). Histograms and mean fluorescence intensities (MFI) of pAMPK within BCL6^{+} Tfh and T-BET^{+} Th1 cells in young (black-outlined curve) and aged (solid red curve) mice 8 dpi. AF647 channel FMO shown in gray. Data compiled from 2 experiments with each data point representing a mouse, bars represent means ± SEM. Tested by two-way ANOVA with Sidak correction for multiple comparisons, *p*-values: ns: not significant.

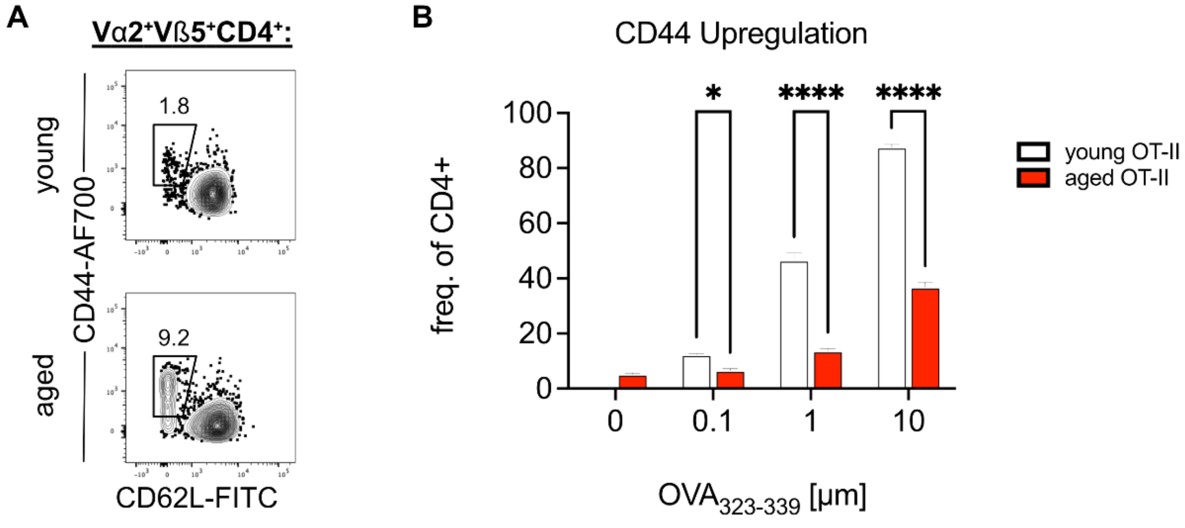

Supplementary Figure 5, in support of Figure 4. A. Frequencies of CD44^{hi}CD62L^{lo} cells within Vα2^{+}Vβ5^{+}CD4^{+} T cells in young (2-3-month-old) and middle-aged (12-month-old) OT-II TCR transgenic mice. **B.** Frequencies of naïve CD4^{+} T cells sourced from young and middle-aged (12-month-old) OT-II mice that have undergone CD44 upregulation in response to *in vitro* co-culture with young splenocytes pre-pulsed with various concentrations of OVAp$_{323\text{-}339}$. Data compiled from 2 experiments with triplicate wells, bars represent means ± SEM. Tested by two-way ANOVA with Sidak correction for multiple comparisons, *p*-values: **** < 0.001, * < 0.05.

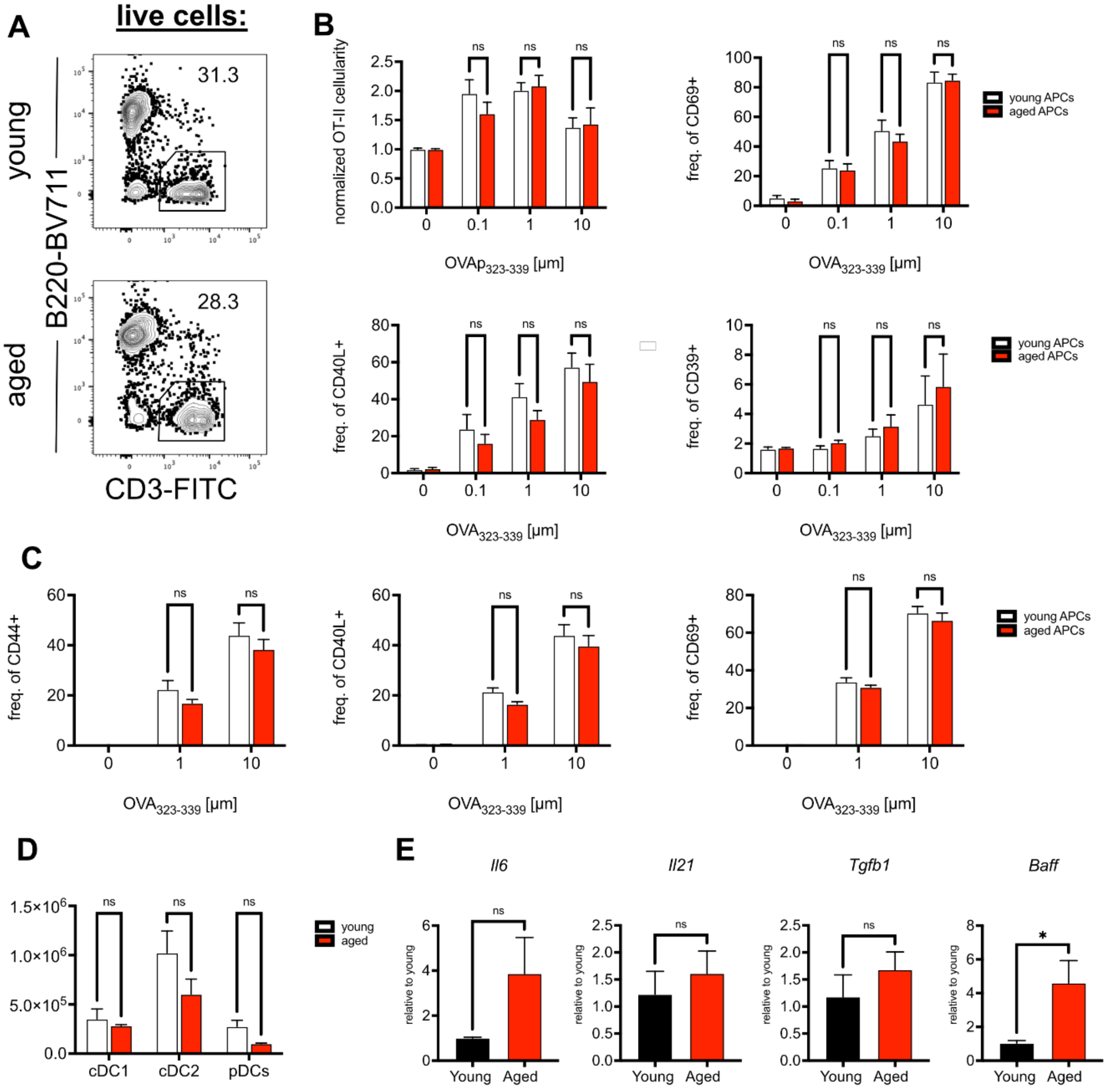

Supplementary Figure 6, in support of Figure 5. A. Proportions of CD3^{+} T cells within bulk spleen cells of young (2-3-month-old) and middle-aged (12-month-old) C57BL/6J mice. **B.** Cellularity of young OT-II CD4^{+} T cells co-cultured with young or middle-aged APCs pulsed with various concentrations of OVAp$_{323\text{-}339}$, normalized to initial cell numbers in each experiment, and OT-II CD4^{+} T cell frequencies that have undergone CD69, CD40L, and CD39 upregulation. Data compiled from 3-7 experiments with the experimental means of triplicate wells, bars represent means ± SEM. Tested by two-way ANOVA with Sidak correction for multiple comparisons, *p*-values: ns: not significant. **C.** Frequencies of young OT-II CD4^{+} T cells that have upregulated

CD44, CD40L, or CD69 after 48-hour co-culture with T/B/NK-depleted splenocytes from young and middle-aged mice pulsed with various concentrations of $OVAp_{323\text{-}339}$. Data compiled from 2 experiments with the experimental means of triplicate wells, bars represent means ± SEM. Tested by two-way ANOVA with Sidak correction for multiple comparisons, *p*-values: ns: not significant. **D.** Cellularity of conventional dendritic cell subsets 1 and 2 (cDC1 and cDC2) and plasmacytoid dendritic cells (pDCs) within young (2-3-month-old) and aged (18-22-month-old) spleens at steady state. **E.** mRNA transcript expression of *Il6*, *Il21*, *Tgfb1*, and *Baff* from young (2-3-month-old) and aged (18-22-month-old) spleens at steady state. Each sample was normalized to expression of *Actb*, and experiments normalized to young specimens. Compiled from 4 mice in each group over 2 experiments. Tested by student t-test, *p*-values: * < 0.05, ns: not significant.

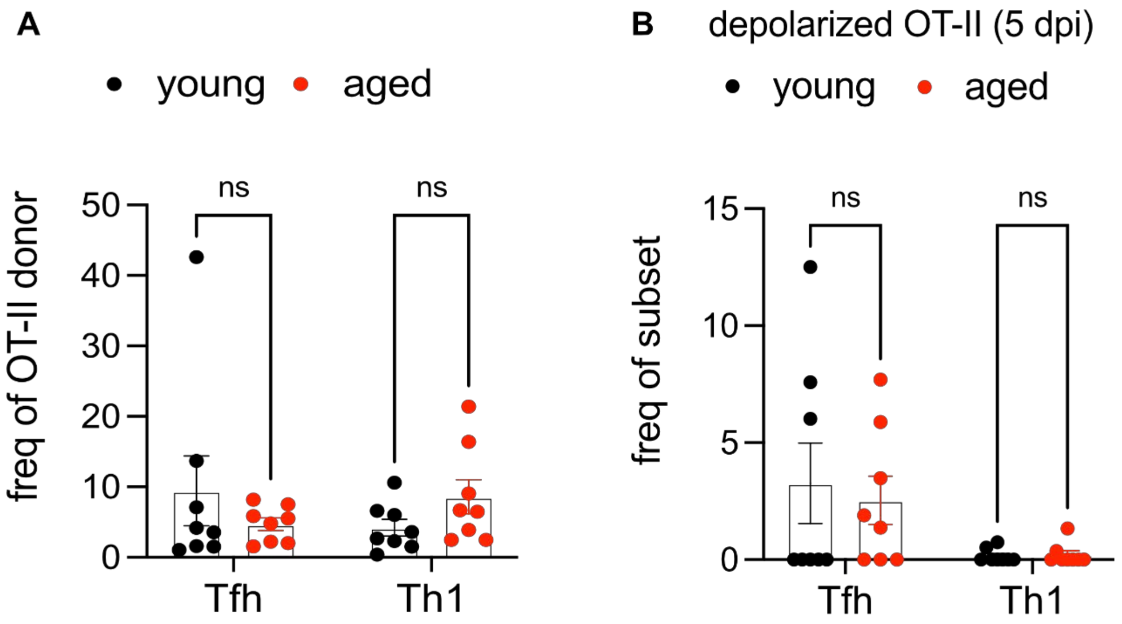

Supplementary Figure 7, in support of Figure 7. A. Frequency of donor OT-II $CD4^+$ T cells that differentiate into Tfh versus Th1 subsets within young (2-3-month-old) and aged (18-22-month-old) mice 5 dpi. **B.** Frequency of depolarized cells within Tfh and Th1 subsets of OT-II donor cells as gated by TMRE-Red^{hi}MTG^{lo}. Data compiled from 2 experiments with each data point representing a mouse, bars represent means ± SEM. Data tested by two-way ANOVA with Sidak correction for multiple comparisons, *p*-values: ns: not significant.

Table 1. Anti-mouse antibodies for flow cytometry

Cell marker	Fluorophore	Clone	Vendor No.
CD3	BV421	17A2	Biolegend 100228
CD3	FITC	17A2	Biolegend 100204
CD3	APCCy7	17A2	Biolegend 100222
CD3	PECy7	17A2	Invitrogen 25-0032-82
CD4	AlexaFluor700	RM4-5	BD Biosciences 557956
CD4	APC	GK15	Biolegend 100412
CD4	BV510	RM4-5	Biolegend 100593
CD4	unconjugated	GK1.5	BioXCell BE0003
CD8	BUV395	53-6.7	BD Horizon 563786
CD8	BV570	53-6.7	Biolegend 100739
CD8	unconjugated	53.6.72	BioXCell BE0004
CD11b	AlexaFluor700	M1/70	Biolegend 101222
CD11b	APC	M1/70	Biolegend 101211
CD11c	BV711	N418	Biolegend 117349
CD11c	APC	N418	Biolegend 117310
CD19	APCCy7	6D5	Biolegend 115530
CD25	AlexaFluor700	PC61	Biolegend 102024
CD39	PEDazzle594	Duha59	Biolegend 143811
CD40	PE	3/23	BD Biosciences 553791
CD40L/CD154	APC	SA047C3	Biolegend 157009
CD40L/CD154	PE	MR1	Biolegend 106505
CD44	AlexaFluor700	IM7	Invitrogen 56-0441-82
CD45.1	PECy7	A20	Biolegend 110730
CD62L	BV605	MEL-14	Biolegend 104437
CD69	APC	H1.2F3	BD Pharmingen 560689
CD69	PE	H1.2F3	BD Biosciences 553237
CD86	BV510	GL-1	Biolegend 105039
B220/CD45R	eFluor450	RA3-6B2	Thermo 48-0452-82
B220/CD45R	BV711	RA3-6B2	Biolegend 103255
B220/CD45R	unconjugated	RA3.3A1/6.1	BioXCell BE006
BCL6	BV421	K112-91	BD Horizon 563363
CXCR5	APC	L138D7	Biolegend 145506
CXCR5	BV650	LI38D7	Biolegend 145517
Fas	APC	SA367H8	Biolegend 152603
FOXP3	APC	FJK-16S	eBioscience 17-5773-80
GL7	eFluor450	GL-7	Invitrogen 48-5902-82
ICOS/CD278	PE	7E.17G9	eBioscience 12-9942-81
ICOS/CD278	PECy7	C398.4A	Biolegend 313519
Ly6C	BV570	HK1.4	Biolegend 128029
MHC-II (I-A/I-E)	PECy7	M5/114.15.2	Biolegend 107629
NK1.1	BV421	PK136	eBioscience 48-5941-1630

NK1.1	unconjugated	PK136	BioXCell BE0036
pAkt308 (Thr308)	unconjugated	D25E6	Cell Signaling 13038T
pAkt473 (Ser473)	unconjugated	polyclonal	Cell Signaling 9271T
pAMPK (Ser79)	unconjugated	polyclonal	Cell Signaling 3661S
PDCA-1/CD317	FITC	927	Biolegend 127007
pS6 (Ser 235/236)	unconjugated	polyclonal	Cell Signaling 2211S
PD-1/CD279	eFluor450	RMPI-30	eBioscience 48-9981-82
PD-1/CD279	FITC	29F.1A12	Biolegend 135213
PSGL-1/CD162	BV605	2PH1	BD Biosciences 740384
T-BET	PECy7	4B10	Biolegend 644823
TCR Vα2	APCCy7	B20.1	Biolegend 127818
TCR Vβ5	FITC	MR9-4	Biolegend 139514
XCR1	APCCy7	ZET	Biolegend 148223
